# Supplementary material for: Transforming solid-state precipitates via excess vacancies
Source: Nat Commun. 2020 Mar 6;11:1248. doi: 10.1038/s41467-020-15087-1 (PMC7060241; doi:10.1038/s41467-020-15087-1)
Supplement: Supplementary file 1 — Supplementary Information [file 41467_2020_15087_MOESM1_ESM.pdf]

Supplementary Information

Transforming Solid-State Precipitates Via Excess Vacancies

Bourgeois et al.

Supplementary Note 1. Additional TEM observations, crystallographic information and DFT calculations

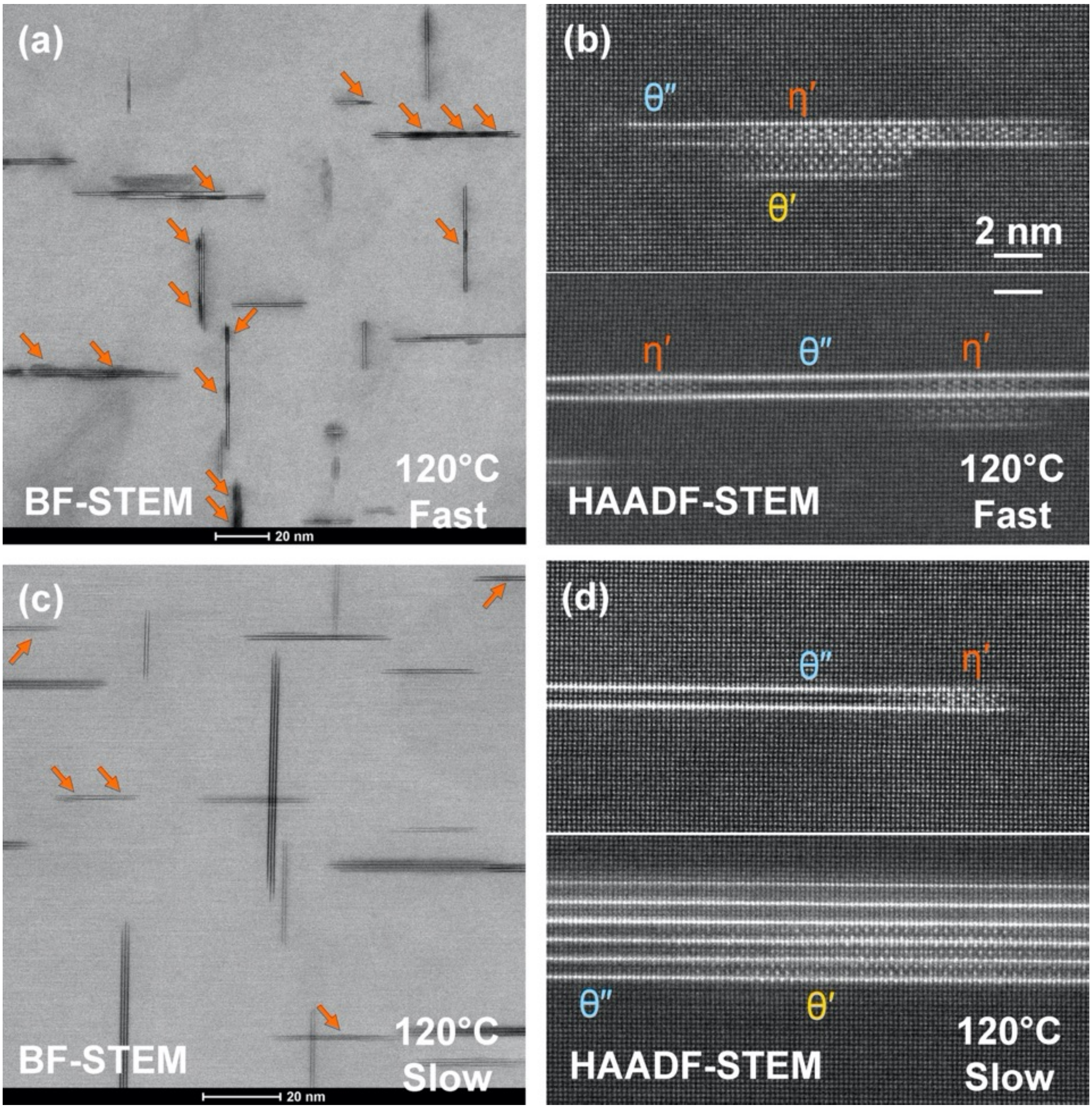

Supplementary Figure 1. Template directed nucleation (TDN) at 120°C. (a)-(b) Sample heated directly from 100°C to 120°C (ramp time ~1 min; hold time at 120°C = 96 min); **(c)-(d)** Sample heated slowly from 100°C to 120°C (2°C ramp every 8-15 min; hold time at 120°C = 15 min). TDN is observed in both cases, *i.e.* regardless of whether the sample is heated rapidly or not. However fewer nuclei are observed in the slowly-heated sample. In **(a)** and **(c)**, arrows point to nucleated precipitates, examples of which are shown at high-resolution in **(b)** and **(d)**. The images were acquired in bright-field (BF) and high-angle annular dark field (HAADF) scanning transmission electron microscopy (STEM) modes.

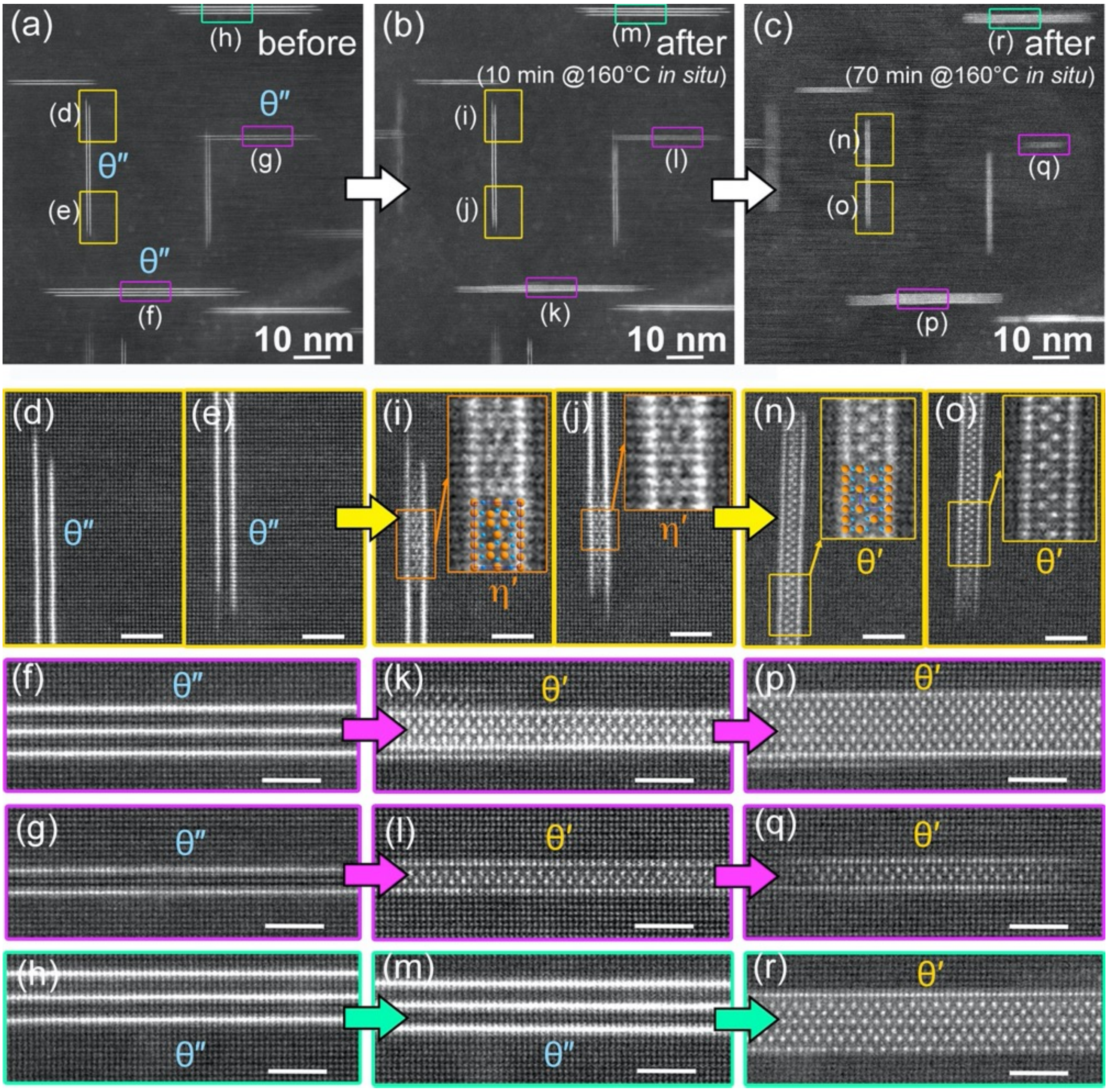

Supplementary Figure 2. TDN at 160°C. This is an expanded version of Fig. 2(a)-(l). The left panels (**(a), (d)-(h)**) show a region and its θ″ precipitates following a conventional bulk heat treatment of 24 h at 160°C, before *in situ* heating. The right panels (**(b), (i)-(m)**) and (**(c), (n)-(r)**) show the same region and precipitates after *in situ* heating for 10 min and 70 min at 160°C, respectively. The θ′ phase and a new phase, η′, have nucleated within the θ″ precipitates. The scale bars in **(d)-(r)** correspond to 2 nm.

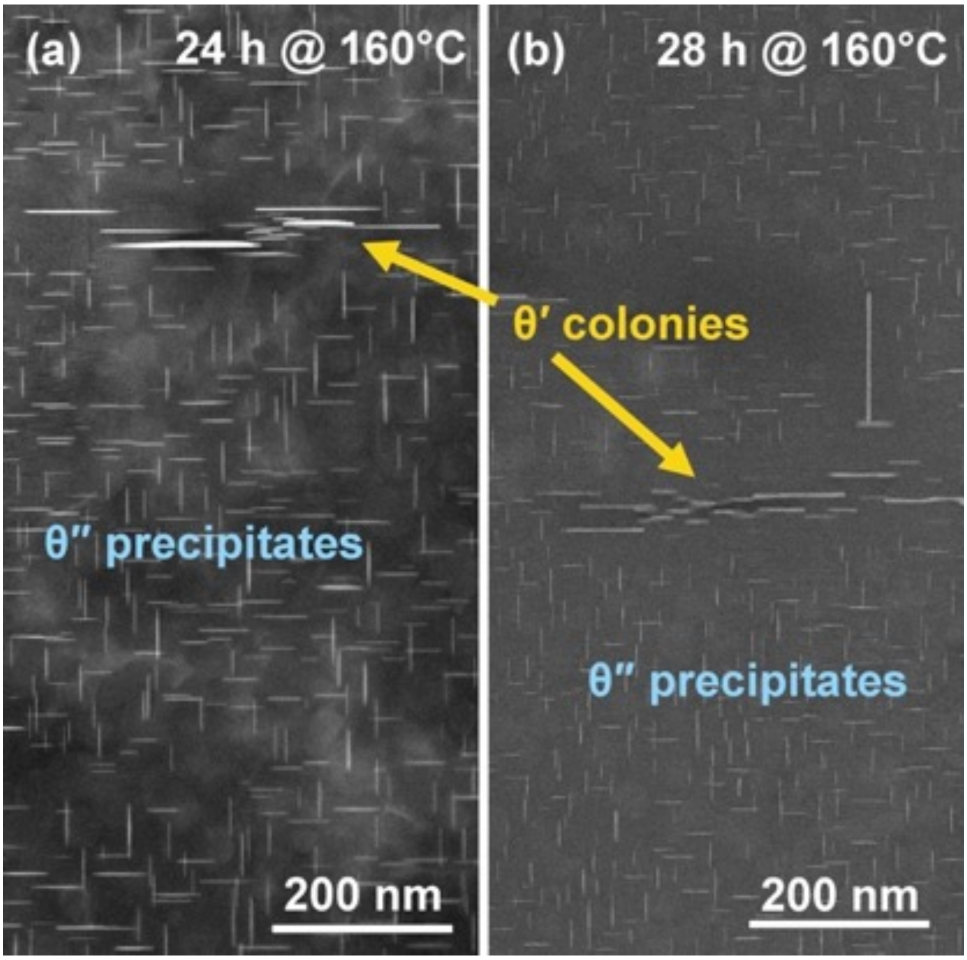

Supplementary Figure 3. Bulk ageing at 160°C of (a) 24 h and (b) 28 h. The additional 3 h ageing results in little difference in microstructure and no TDN.

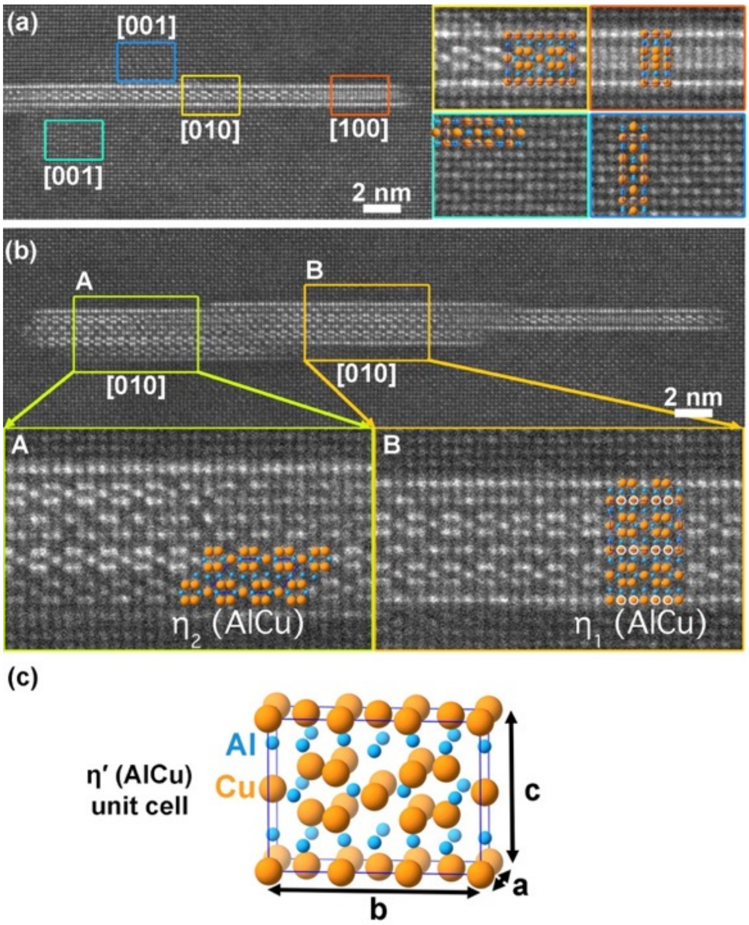

Supplementary Figure 4. The precipitate phase η′ has a crystal structure based on the bulk thermodynamically stable phases η_1 and η_2. (a) displays a HAADF-STEM image of several η′ nuclei within a θ″ precipitate as well as a nucleus in plan view, which constitute three variants of the η′ phase (see enlargements with the crystal structure overlaid). **(b)** shows a thicker precipitate containing several η′ variants exhibiting different stacking configurations. These stackings can locally resemble the η_2 or η_1 bulk phases (see enlargements A and B, respectively). Regions of η_1 structure can be regarded as multiple unit cells of the orthorhombic η′ structure with fractional occupancies of some Cu atoms (Cu3 in Supplementary Table 1). We solved the crystal structure in two steps. Firstly, we developed a preliminary model by starting with a pure FCC Al structure and replacing Al atoms by Cu atoms based on the three variant structures observed by HAADF-STEM (a). Secondly, the atomic positions and lattice parameters of this preliminary model were relaxed using Density Functional Theory (DFT). The crystal symmetry and DFT-refined atomic positions are provided in Supplementary Table 1. **(c)** presents one unit cell of η′ phase viewed near [100]. Strictly speaking, the η′ structure just described only applies to the single-unit-cell thick precipitates shown in **(a)** and Fig. 2(g)-(h). These are two-dimensional crystals, with no periodicity in the c direction. Note that STEM images resembling the η′ phase were reported very recently [1,2]; however the proposed crystal structure was incorrect.

Supplementary Table 1. Crystallographic information for the η′ precipitate phase (AlCu) following DFT relaxation. The crystal data provided here assumes a three-dimensional crystal only for the convenience of building the crystal structure.

Space group: *Cmmm*
Lattice parameters: *a*=4.12 Å, *b*=12.16 Å, *c*=8.80 Å

Atomic coordinates and site occupancies

Atom	*x/a*	*y/b*	*z/c*	*Occ.*
Cu1	0.5	0.819	0.349	1
Cu2	0	0.5	0.5	1
Cu3	0.5	0.841	0	1
Cu4	0.5	0.5	0.5	1
Al1	0	0.837	0.171	1
Al2	0	0.5	0.200	1
Al3	0	0.881	0.5	1

Supplementary Table 2. Energetics of the different phases as calculated by DFT. E_f is the formation energy, E_{Cu} the defect energy of Cu in Al, and E_f^{Cu} the formation energy relative to E_{Cu} (see Methods, Main Manuscript). The supercells used for the precipitate structures are shown in the Supplementary Information (Supplementary Figure 5). The uncertainties are ±2 meV. GP interface refers to an interface with interstitial Cu atoms, thus corresponding to a Cu monolayer as in a Guinier-Preston zone.

Defect	E_{Cu} *(meV)*	E_f^{Cu} *per atom (meV)*
1 Cu atom	-122	0
Phase	E_f *per atom (meV)*	E_f^{Cu} *per atom (meV)*
Bulk θ″	-90	-59
Bulk θ′	-180	-140
Bulk η_1	-196	-135
Bulk η_2	-216	-155
1$c_{\theta''}$-thick θ″ precipitate	-78	-47
1.5$c_{\theta'}$-thick θ′ precipitate	-149	-109
1.5$c_{\theta'}$-thick θ′, GP interface	-166	-114
2$c_{\theta'}$-thick θ′ precipitate	-152	-111
2$c_{\theta'}$-thick θ′, GP interface	-177	-126
1$c_{\eta'}$-thick η′ precipitate	-182	-121
1c_{η}-thick η_2 precipitate	-170	-115

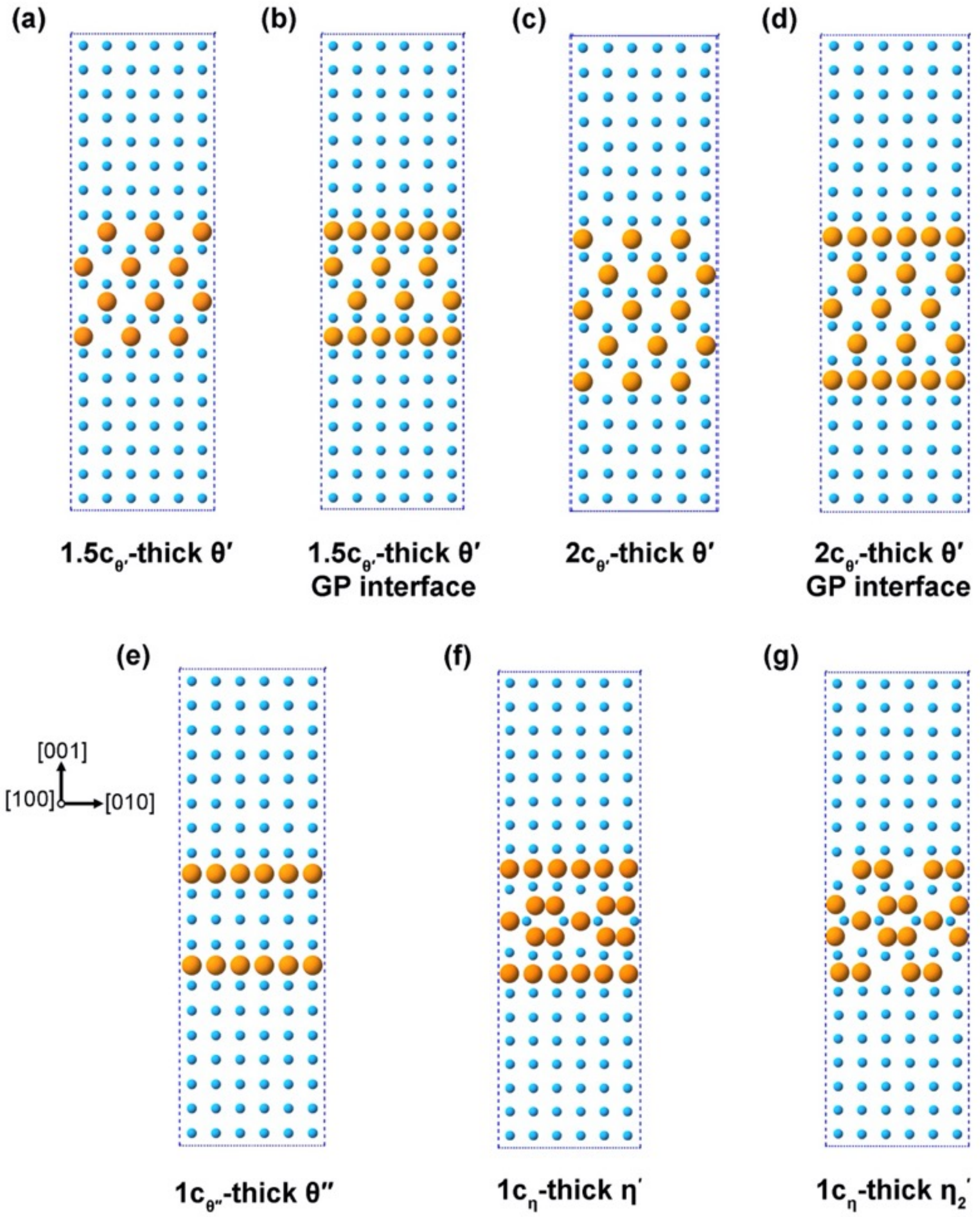

Supplementary Figure 5. Supercells used for the DFT calculations (Supplementary Table 2). **(a)** 1.5$c_{\theta'}$-thick θ′ precipitate, **(b)** 1.5$c_{\theta'}$-thick θ′ precipitate with GP zone coherent interface, **(c)** 2$c_{\theta'}$-thick θ′ precipitate, **(d)** 2$c_{\theta'}$-thick θ′ precipitate with GP zone coherent interface, **(e)** θ″ precipitate, **(f)** 1$c_{\eta'}$-thick η′ precipitate (based on the bulk phase η_1 – see Supplementary Table 1), **(g)** 1$c_{\eta'}$-thick η_2' precipitate (based on the structure of the bulk phase η_2). The crystallographic directions shown apply to all phases and supercells.

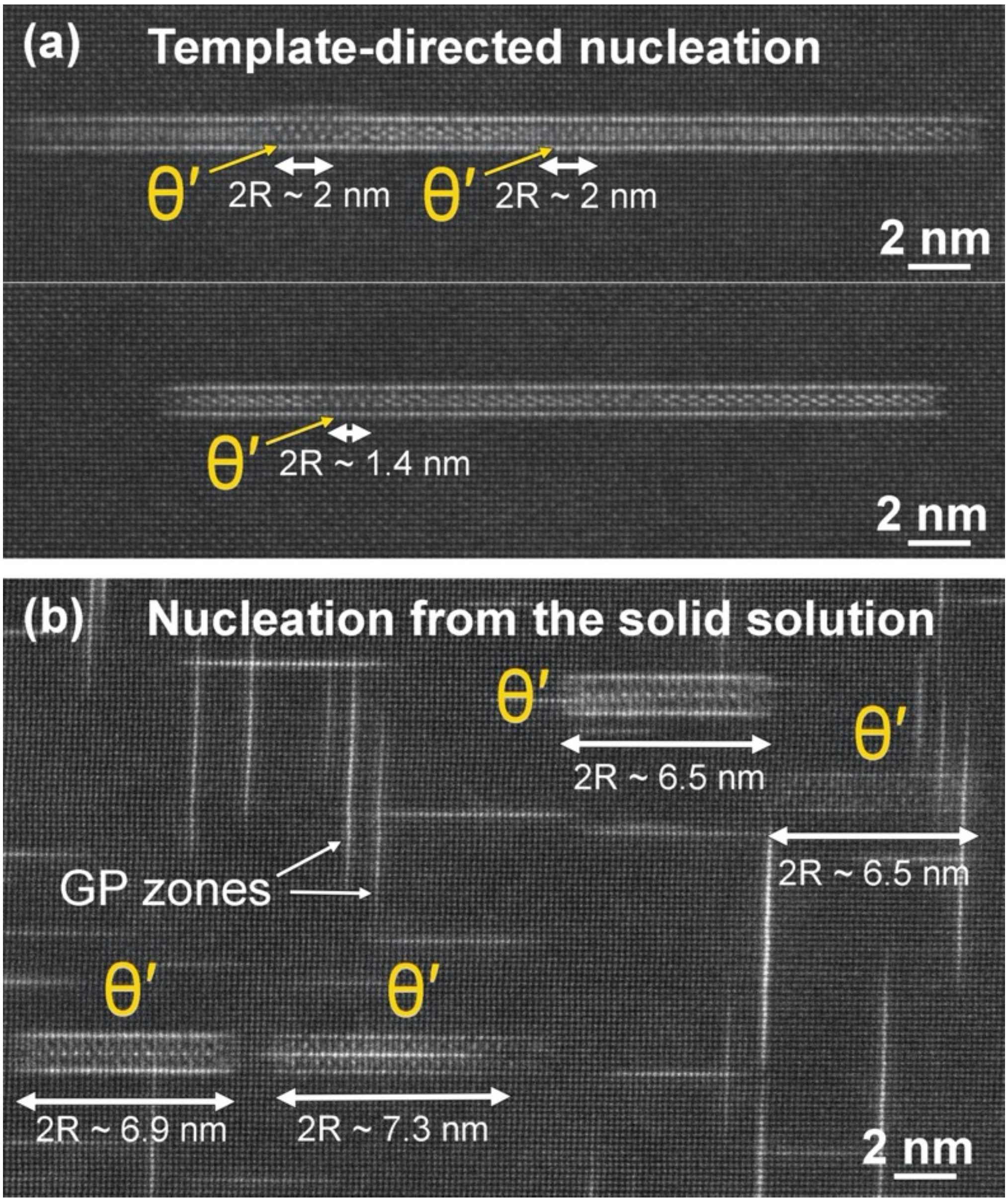

Supplementary Figure 6. Estimation of the critical radius of nucleation, *R, of θ′ phase.** In Classical Nucleation Theory, R^* corresponds to the maximum of the free energy curve (Fig. 4(c)), where 50% of nuclei will survive and grow. The smallest experimentally observed precipitate radius in the earliest stages of precipitation and in conditions of slow kinetics should therefore provide a good estimate of R^*, **(a)** in TDN at 150°C (18 min) and **(b)** in a conventional ageing treatment at 100°C (2 days). It is around 1 nm for TDN at 150°C **(a)** and 3 nm for nucleation from the solid solution in the bulk **(b)**. In TDN most θ′ precipitates are found to grow very fast so that it is very difficult to capture the nucleation process. The examples of small θ′ precipitates shown in **(a)** are the smallest ones found for TDN.

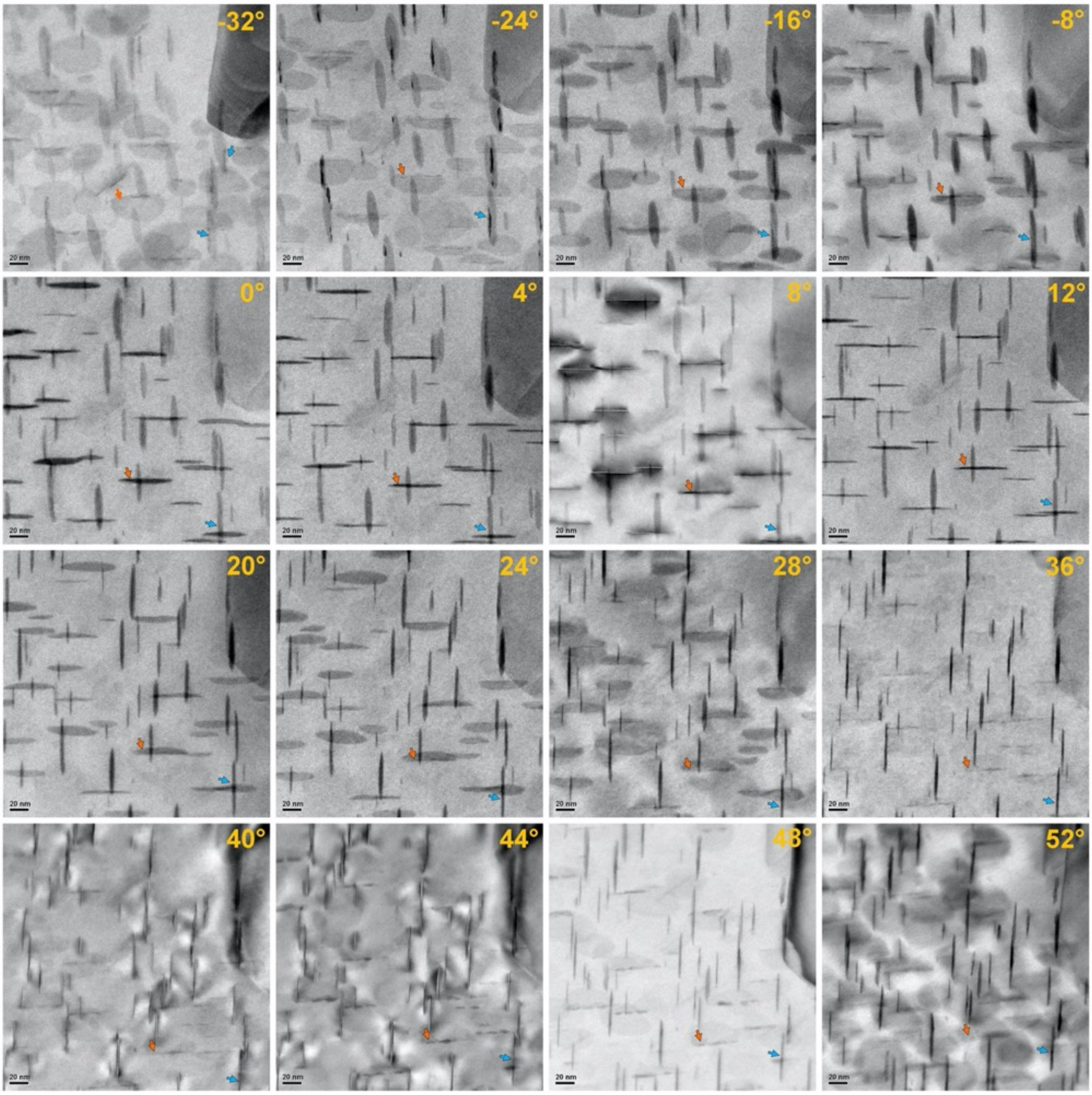

Supplementary Figure 7. Demonstration that TDN takes place just below the sample surface. BF-STEM tilt series revealing that TDN occurred near the cut surface of θ″ precipitates. The sample's tilt angle is shown in the upper right corner of each image. The blue and orange arrows each point to the same θ″ precipitate viewed at different angles. The precipitate side cut by the sample surface is evident for the orange-arrowed case, on which nuclei (darker contrast) are clearly visible.

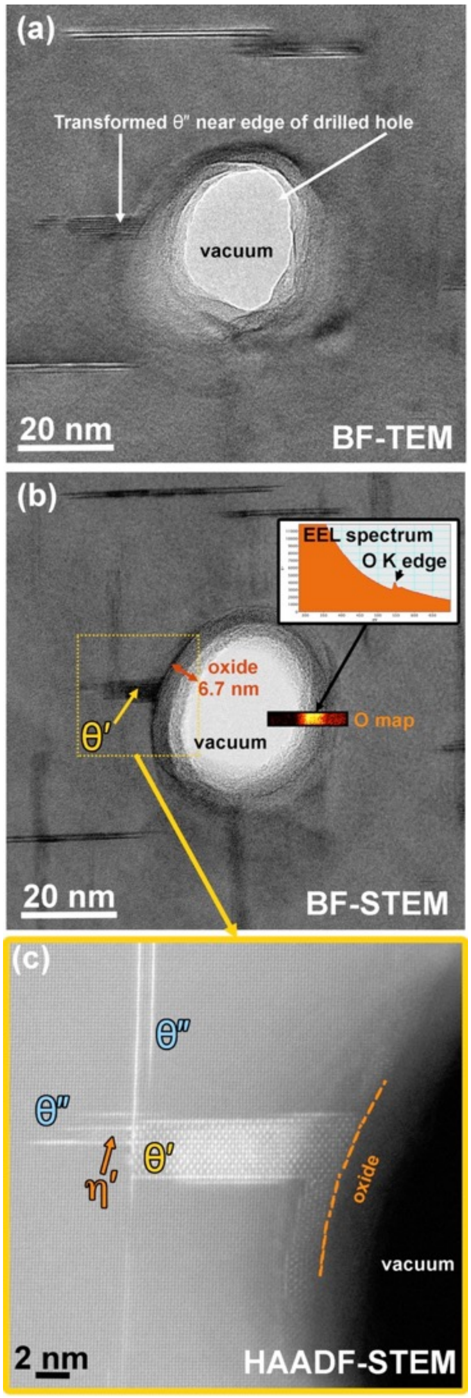

Supplementary Figure 8. Demonstration that TDN takes place at the interface between the aluminium matrix and a native oxide at least 6 nm thick. (a) A ~30 nm wide hole was drilled using a focused electron beam in a TEM specimen prepared from a bulk treated alloy (24 h ageing at 160°C). The specimen remained in the high vacuum of the microscope (~10^{-5} Pa) for ~2 hours before undergoing *in situ* heating for 10 min at 160°C. The bright field TEM image (a) reveals nucleation slightly away from the edge of the hole, just after discontinuing *in situ* heating, already suggesting the presence of an oxide layer several nanometres thick having formed inside the microscope. The data presented in (b) and (c) was collected a week later in a different microscope. **(b)** Electron energy loss (EEL) spectroscopy confirmed the region between transformed precipitate and vacuum to be an oxide. **(c)** HAADF-STEM imaging shows the oxide is amorphous and the transformed precipitate contains both η′ and θ′. The thicker θ′ precipitate probably arises from its close proximity to three surfaces rather than two, namely the sample surfaces and the hole's edge.

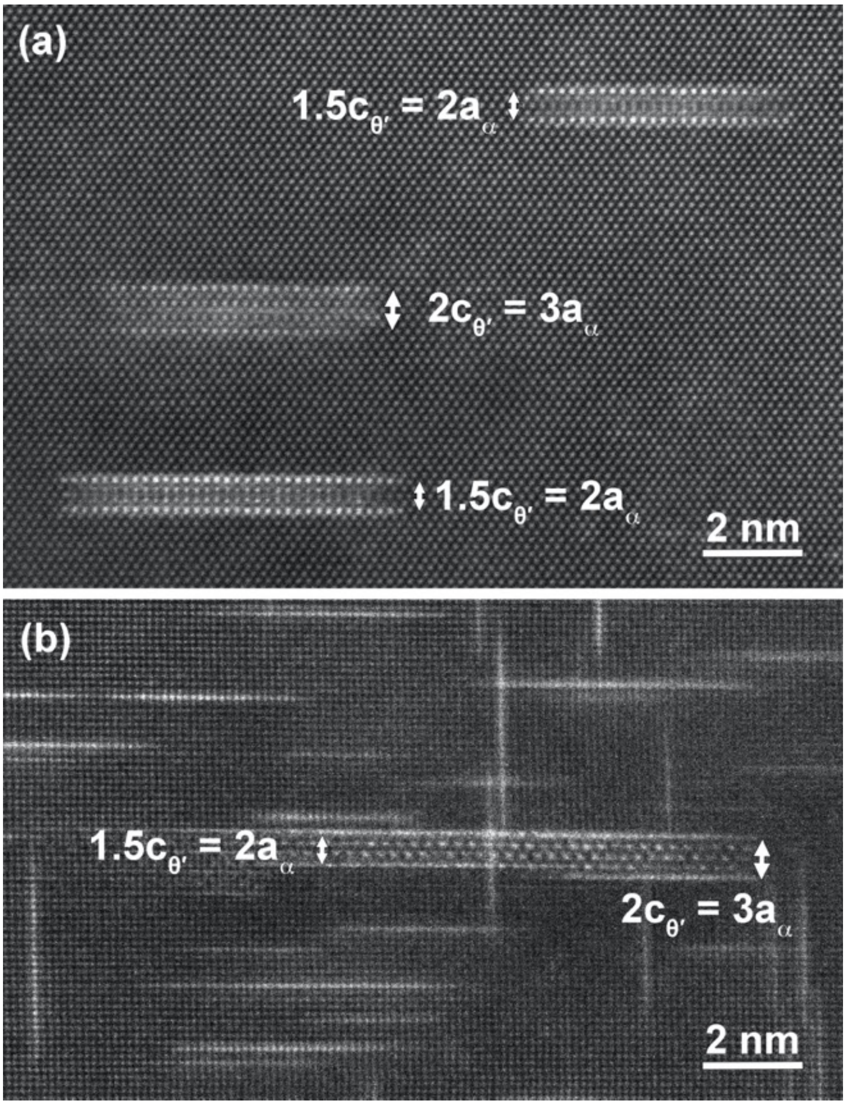

Supplementary Figure 9. Existence of 1.5c$_{\theta'}$-thick θ′ in conventionally heat treated alloys, here at 100°C following (a) 7 days or (b) 2 days, as viewed along (a) <110> or (b) <100>. The middle precipitate in **(a)** does not reveal its internal structure, most likely because it lies deeply inside the matrix.

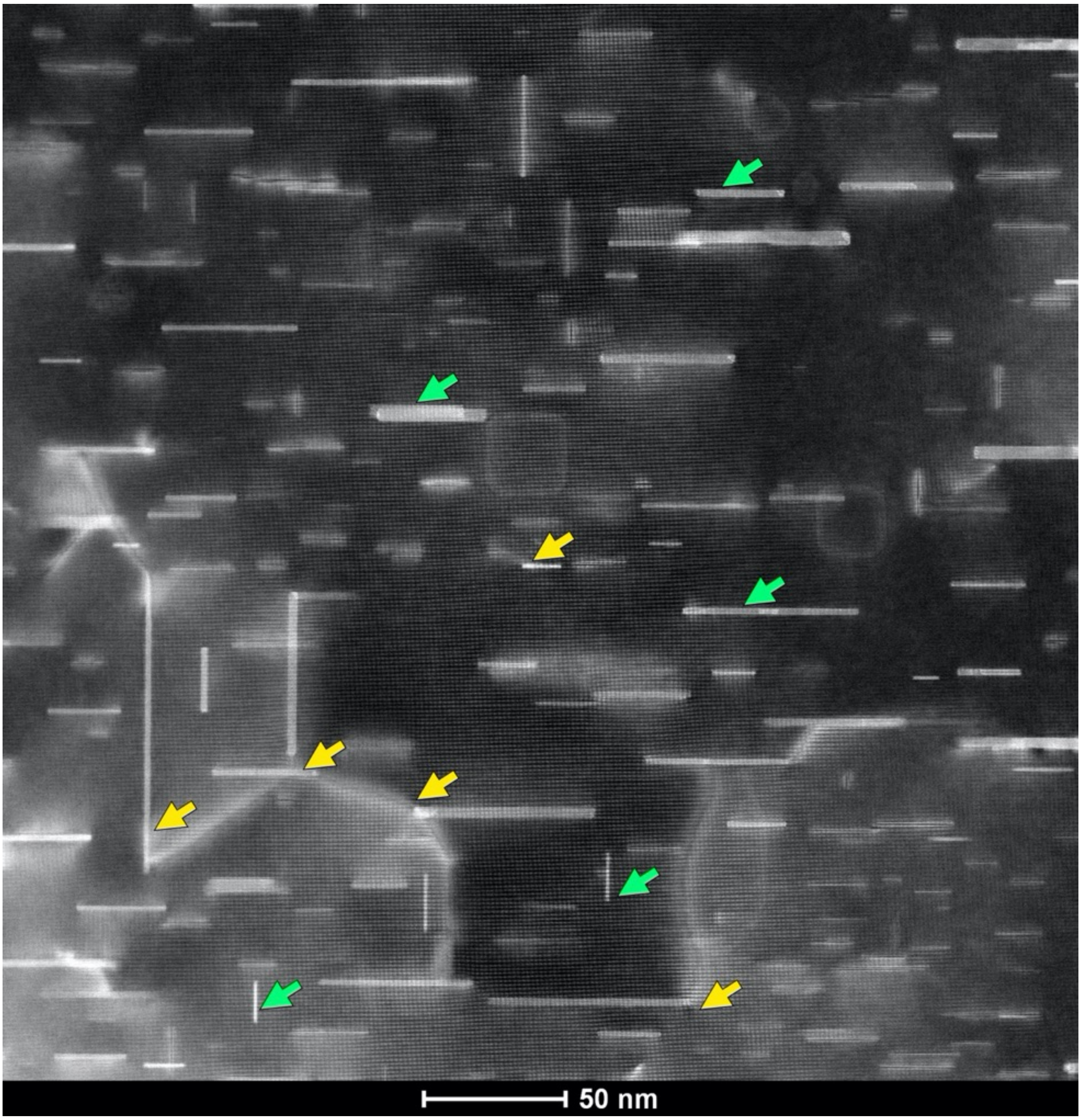

Supplementary Figure 10. Many precipitates transformed from θ″ did not form directly on dislocations introduced by deforming the sample. The microstructure of a sample aged 5 h at 160°C, deformed 5% and aged again at 160°C for 3 h shows the presence of dislocations and precipitation on those dislocations (yellow arrows) but also many precipitates not associated with dislocations (green arrows). The imaging conditions used were low-angle annular dark field, which enhances strain contrast. Note that the lattice contrast visible in the image arises from aliasing between the crystal lattice and the STEM image scan (2048x2048).

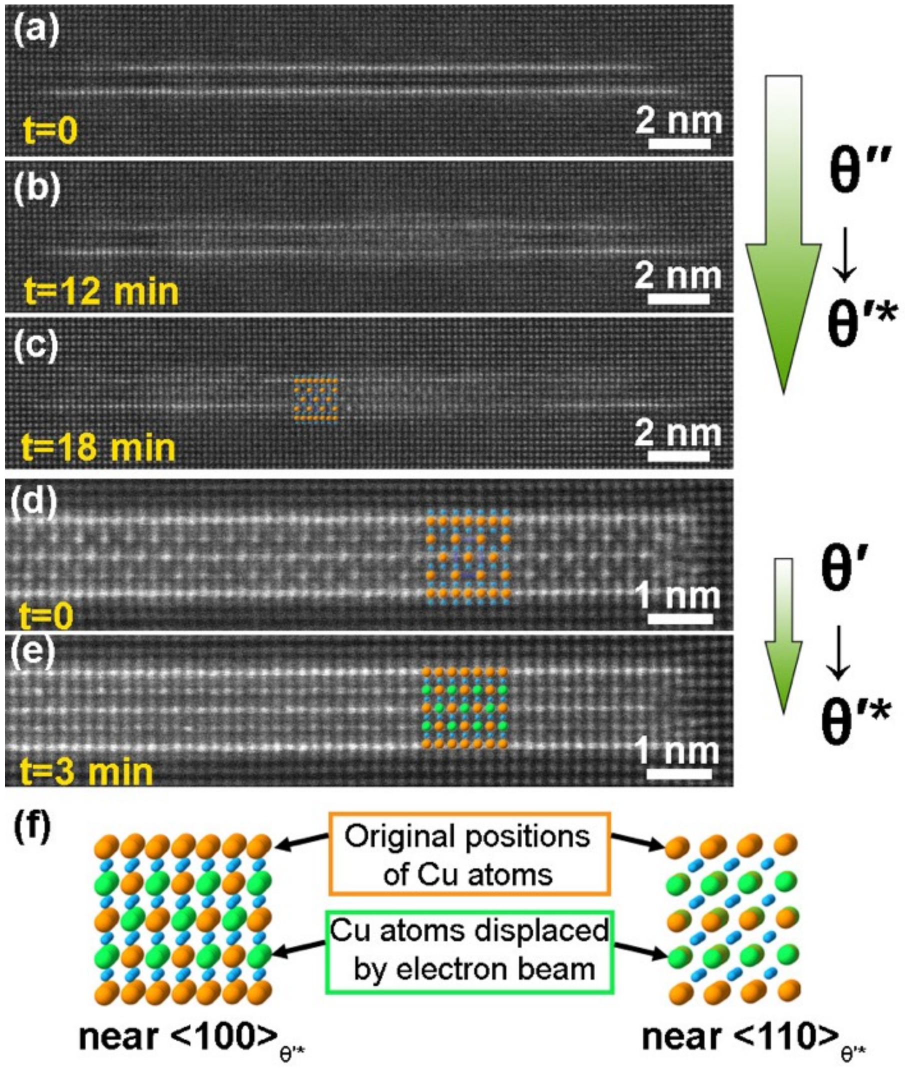

Supplementary Figure 11. TDN of a disordered analogue of θ′, θ′*, is caused by a 300 keV electron beam interacting with θ″; θ′* is closely related in structure to beam-damaged θ′. (a) shows a pristine θ″ precipitate viewed along <100>. **(b)** and **(c)** show the same θ″ precipitate following exposure to the electron beam in scanning mode for 12 and 18 min, respectively, by scanning the incident beam at high magnification over a region ~10 ×10 nm^2 in size. Regions of θ′ structure are visible, but considerable disorder is apparent. The transformed **θ′*** is very similar to beam-damaged **θ′** (see **(d)** and **(e)**). A model for θ′* is presented in **(f)** along <100> and <110>, where some Cu atoms are displaced into interstitial positions (green). Clearly θ′* will look identical to θ′ when viewed along <110> (in accordance with the observations shown in Fig. 5(i)-(j)) but not so when viewed <100>, as confirmed in **(b)-(c)** and **(e)**.

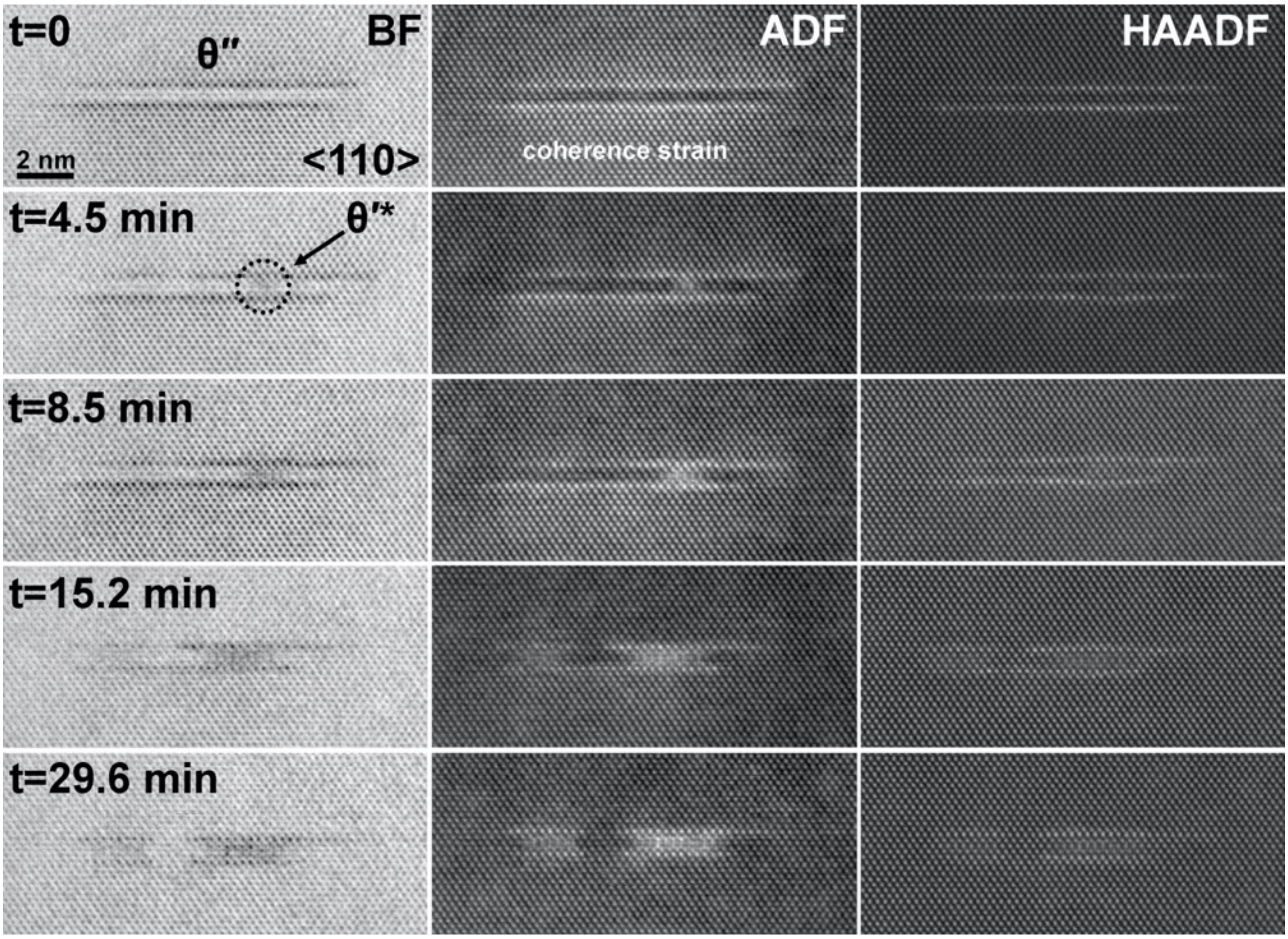

Supplementary Figure 12. No vacancy loops were observed associated with the θ″-to-θ′* transformation. Bright-field (BF) and annular dark field (ADF) STEM images showing the electron beam induced transformation (see Fig. 6) revealed no dislocation contrast. These image modes are very sensitive to structural changes, as evident from the clear detection of a nucleus 1 nm in diameter after 4.5 min of electron beam exposure. Note the reduction in coherence strain as the coherent precipitate θ″ transform to the semi-coherent phase θ′*.

Supplementary Note 2. Atomic scale mechanisms of the θ″-to-θ′ transformation

We propose the mechanisms shown in Supplementary Figure 13 for the θ″-to-θ′ transformation, incorporating vacancies in both a kinetic and thermodynamic capacity.

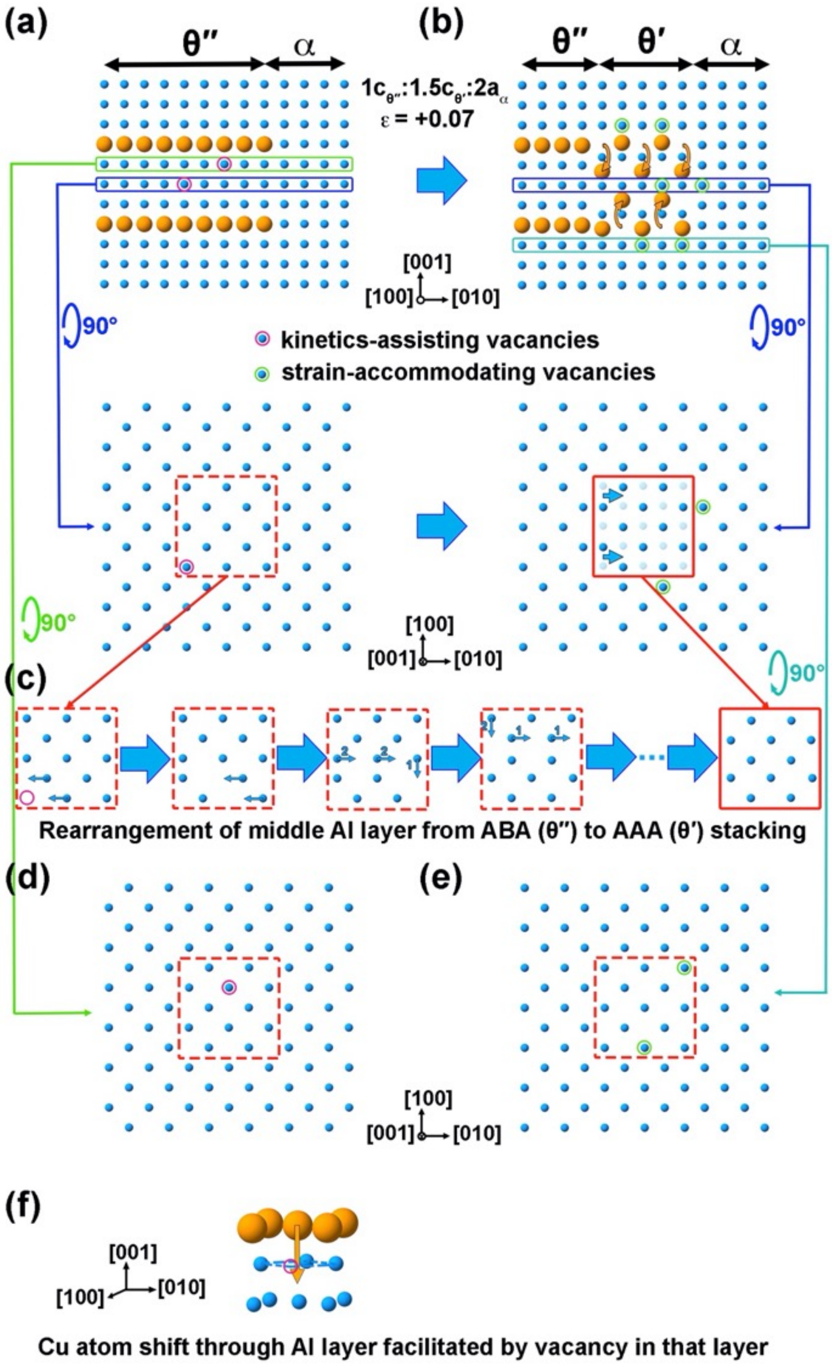

Supplementary Figure 13. Atomic scale mechanism proposed in this work for the θ″-to-θ′ transformation. As in earlier models ([3-4], see Supplementary Figure 15), vacancies are crucial. However they are not structural. A small number of vacancies (shown as pink circles in **(a)**, **(c)**, **(d)** and **(f)** in the θ″ structure) play a kinetic role by facilitating the stacking change **(c)** and lowering the barrier for Cu atomic shifts **(f)**. Additional vacancies (shown as green circles in **(b)** and **(e)** in the θ′ structure) surrounding the nucleus will relieve misfit strain and therefore lower the thermodynamic barrier to nucleation.

The atomic scale mechanisms proposed are supported by semi-empirical Deep Neural Network Potential (DNNP) simulations and DFT – see Supplementary Figure 14 and Supplementary Table 3. In particular, our DNNP simulations show that up to 3 vacancies per coherent interface of a θ′ precipitate 1 nm in radius will be favoured to segregate, or about 3 vacancies per 20 surface atoms. A similar finding was obtained by DFT simulations for a semi-infinite precipitate: a vacancy close to the coherent interface (position 2 in Supplementary Figure 14(e)) will experience a slightly lower formation energy compared to a vacancy far away (position 3) – see Supplementary Table 3. Position 1 was found to be unfavourable, a result that differs from DNNP.

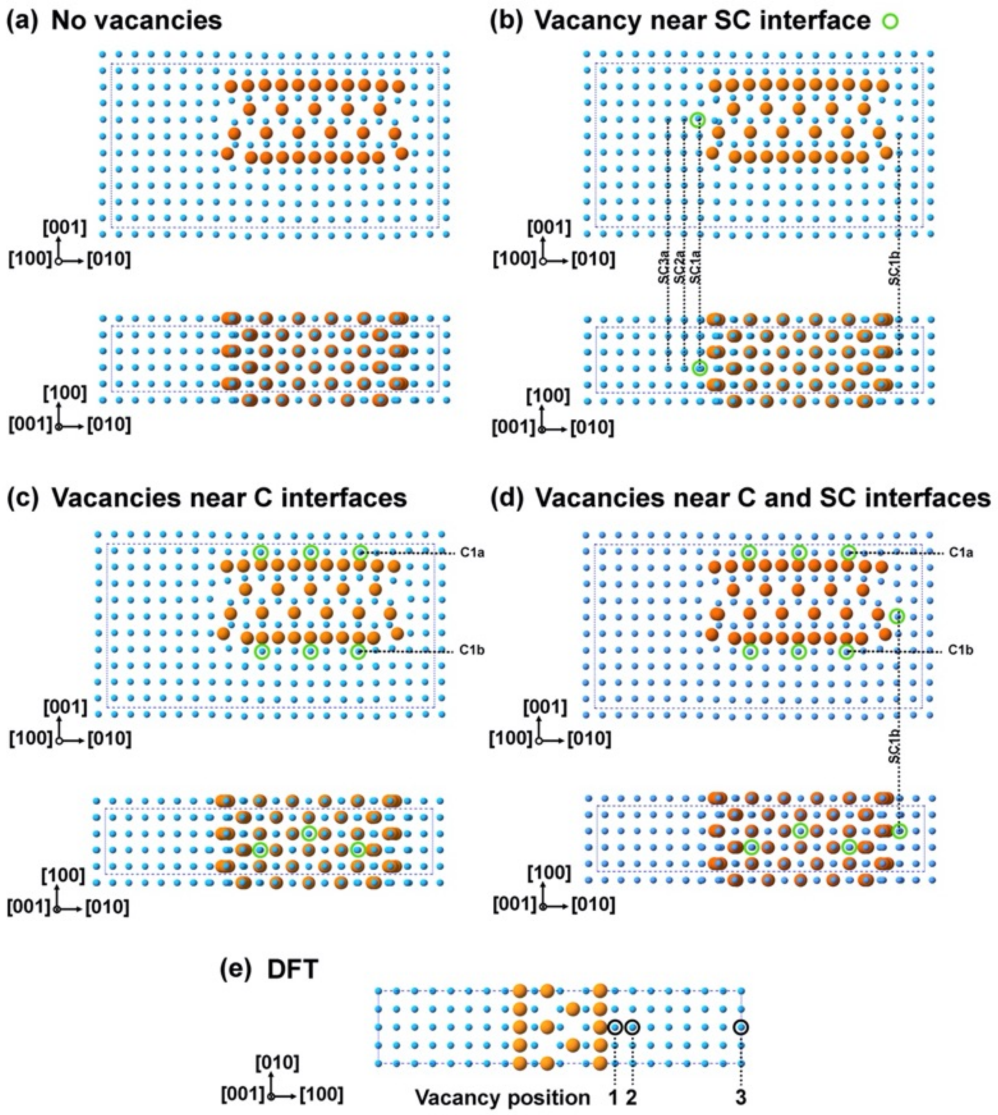

Supplementary Figure 14. Supercells (dashed blue lines) used in simulations of the energetics of a 1.5$c_{\theta'}$-thick θ′ precipitates surrounded with different vacancy populations, via (a)-(d) Deep Neural Network Potentials (DNNP) and (e) Density Functional Theory (DFT). In **(a)-(d)** the θ′ precipitate is 2 nm wide along [010] and infinite along [100]. In **(e)** the θ′ precipitate is infinite along both [100] and [010]. C and SC refer to coherent and semi-coherent. According to DNNP all three vacancy-containing systems (**(b)-(d)**) result in a lower energy compared with the case of nucleus far from vacancies shown in **(a)** (see Supplementary Table 3). Similarly, DFT shows that a vacancy has a lower formation energy close to the coherent interface (Supplementary Table 3).

Supplementary Table 3. Vacancy (V) formation energies E_f at 0K as calculated by DNNP or DFT using the supercells shown in Supplementary Figure 14. C and SC stand for coherent and semi-coherent interface, respectively.

Configuration (DNNP)	*Supplementary Figure 14 Ref.*	E_f *per vacancy (eV)*	$E_f - E_f^V$ *(eV)*
1V in bulk Al	--	0.65 = E_f^V	0
1V at SC1a	(b)	0.28	-0.37
1V at SC2a	--	0.39	-0.26
1V at SC3a	--	0.63	-0.02
1V at SC1a + 1V at SC1b	--	0.73	0.08
1V at C1a	--	0.59	-0.06
3V at C1a	--	0.58	-0.07
3V at C1a + 3V at C1b	(c)	0.61	-0.04
5V at C1a + 5V at C1b		0.67	+0.02
3V at C1a + 3V at C1b + 1V at SC1a	(d)	0.63	-0.02
Vacancy position (DFT)	*Supplementary Figure 14 Ref.*	E_f *per vacancy (eV)*	$E_f - E_f^V(DFT)$ *(eV)*
1	(e)	0.829	0.29
2	(e)	0.555	-0.04
3	(e)	0.593 = $E_f^V(DFT)$	0

According to these simulations, vacancies will also tend to segregate at one side of the semi-coherent interface. However, without an accurate model of the semi-coherent interface it is not possible to obtain more specific information about where such vacancies might prefer to segregate.

All atomistic models proposed before the present work for the θ″-to-θ′ transformation [3-4] required structural vacancies to accomplish the change in stacking from ABA to AAA (see Fig. 2(p)). However, and this is a point only made in Dahmen and Westmacott [3], once incorporated into the newly formed nucleus, these vacancies must be ejected for the nucleus to have the correct volume and for continuity between precipitate and matrix. In other words, there is no net vacancy volume absorbed or ejected. This is shown in Supplementary Figure 15. Our experiments (Supplementary Figure 12) revealed no such vacancy loops. Furthermore, the strain associated with such loops should make models (a) and (b) energetically more costly than our model (c).

Another factor favouring our model is its requirement of far fewer vacancies than in previous models [3-4], as can be seen in Supplementary Table 4. The number of vacancies required for different processes, including the three competing atomic mechanisms of the θ″-to-θ′ transformation, is shown for a θ′ nucleus 1 nm in radius. Such a nucleus will require a minimum of ~12 vacancies according to the present model: 3 for the structural transformation and ~9 for strain accommodation and reduction of semi-coherent interfacial energy – see Supplementary Figures 12-13. Once the structural transformation is completed, the ~3 vacancies can be used for partial strain / interfacial accommodation. In contrast, earlier models [3-4] require ~140 vacancies each: ~72 for each of the two vacancy loops in Ref. [3] and ~36 for each of the four half-filled Cu (002) planes in Ref. [4] (see

Supplementary Figure 15). Even assuming some of these vacancies (~10) remain to act as strain relief and lowering of the interfacial energy, the majority of the ~140 vacancies will still need to be emitted, as noted above. These excess vacancies may be ejected as vacancy loops (as suggested in Ref. [3]) or continue the θ″-to-θ′ transformation process. Ultimately, the vacancies will have to be emitted once the entire precipitate has transformed.

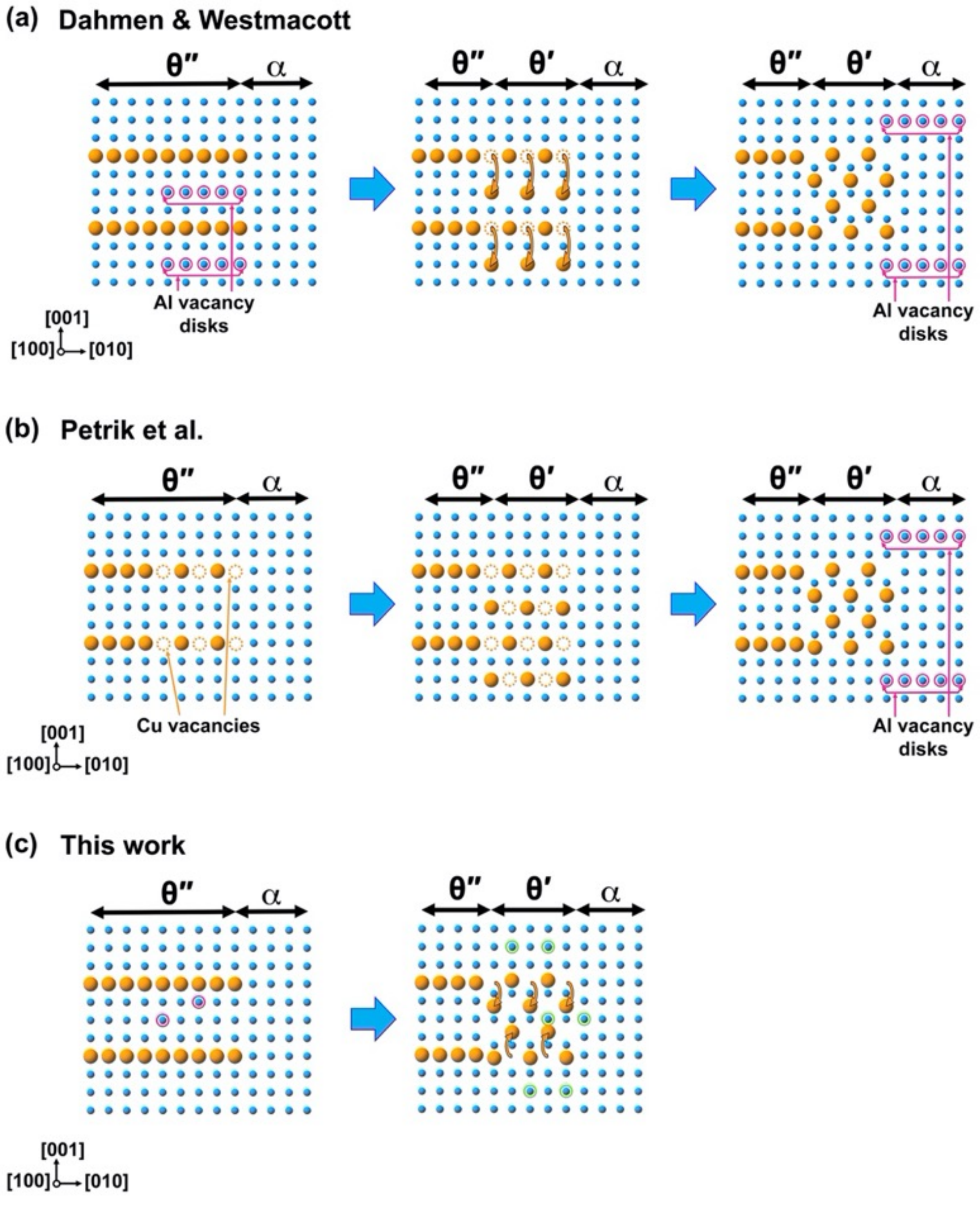

Supplementary Figure 15. Past and present atomistic models for the θ″-to-θ′ transformation. All models, including this work, require vacancies. In **(a)** (Dahmen & Westmacott [3]) and **(b)** (Petrik *et al.* [4]), vacancies aggregate either on (002) Al planes or on the (001) Cu planes of θ″, leading to the required change in Al plane stacking for the nucleation of θ′. However, these structural vacancies must be emitted to obtain the correct volume for the nucleus and restore continuity with the matrix. Our model **(c)** does not use structural vacancies for the transformation, as shown in detail in Supplementary Figure 13.

Supplementary Table 4. Number of vacancies (NV) associated with different processes for a $1.5c_{\theta'}$-thick θ′ nucleus 1nm in radius. I sites refer to interstitial Cu sites at the coherent interfaces of θ′ precipitates.

**θ″-to-θ′ transformation**		
Ref.	*minimum NV required*	*NV to be ejected*
This work	12	0
Dahmen and Westmacott [3]	~140	~130
Petrik *et al.* [4]	~140	~130

**NV potentially available at 160°C**		
Supersaturation Vss	*ln(Vss)*	*NV per θ″ precipitate (after 24h ageing)*
1 (thermal equilibrium)	0	0.05 (i.e. 1/20)
20	3	1
40	3.7	2
200	5.3	10
2000	7.6	100
3000 (max for quenching from 525°C)	7.9	150

**NV associated with Cu diffusing to I sites of θ′ nucleus**	~70

It is instructive to compare the requirement in vacancy numbers for the different models of the θ″-to-θ′ transformation, with vacancy numbers arising from different vacancy supersaturations – see Supplementary Table 4. It can be seen that significant vacancy supersaturations will be needed for all three models, but particularly so for Dahmen & Westmacott and Petrik *et al.*'s models [3-4].

Once θ′ has nucleated on θ″, Cu atoms will diffuse to the vacant interstitial sites (I sites in Supplementary Table 4) at the coherent interface, as observed experimentally. This will create additional vacancies numbering ~70 for a nucleus 1 nm in radius. With the reasonable assumption that those Cu atoms originate from neighbouring regions of the template precipitate θ″, those regions will adopt a structure similar to that examined by Petrik *et al.* [4] and depicted on the leftmost panel of Supplementary Figure 15(b). This structure was shown to be stable according to DFT [4] and may persist, or may eventually release its vacancies and participate in the growth of the θ′ nucleus. It should be noted, however, that these vacancies within Cu (002) planes are likely not to be as free moving as vacancies in the Al matrix, because they cannot be filled with Al atoms. The detail of how such vacancies may affect growth (lengthening, and possibly thickening) of the nucleus awaits further studies.

Supplementary Note 3. Classical Nucleation Theory calculations

These calculations consider nucleation of the θ′ phase, either directly from a supersaturated solid solution (homogeneous nucleation) or on a pre-existing coherent θ″ phase. Nucleation of the η′ phase was not investigated because there exists little thermodynamic data on this metastable phase.

Supplementary Note 3.1. Nucleation of θ′ phase directly from solid solution

According to Classical Nucleation Theory (CNT), the total energy ΔG associated with nucleation of a precipitate of volume V, surface area A and interfacial energy γ can be written as

$$\Delta G = V(\Delta G_{chem} + \Delta G_{el}) + A\gamma, \qquad (1)$$

where ΔG_{chem} is the change in the chemical component of the volume free energy (or chemical free energy) associated with solute atoms in the matrix forming a nucleus, V is the volume of the nucleus, and ΔG_{el} is the elastic energy associated with nucleation. We assume a pancake-shaped precipitate of radius R and thickness t (see Supplementary Figure 16(a)), where the broad face is coherent with the matrix with an interfacial energy γ_c and the rim is semi-coherent with an interfacial energy γ_{sc}. This yields

$$\Delta G = V(\Delta G_{chem} + \Delta G_{el}) + 2\pi R^2\gamma_c + 2\pi Rt\gamma_{sc} \qquad (2)$$

Three thicknesses of θ′ precipitates were considered, $t = 1c_{\theta'}$, $t = 1.5c_{\theta'}$ and $t = 2c_{\theta'}$, where $c_{\theta'}$, is the c lattice parameter of θ′; these thicknesses correspond to the three thinnest configurations possible for a θ′ nucleus – see Supplementary Figure 16(b).

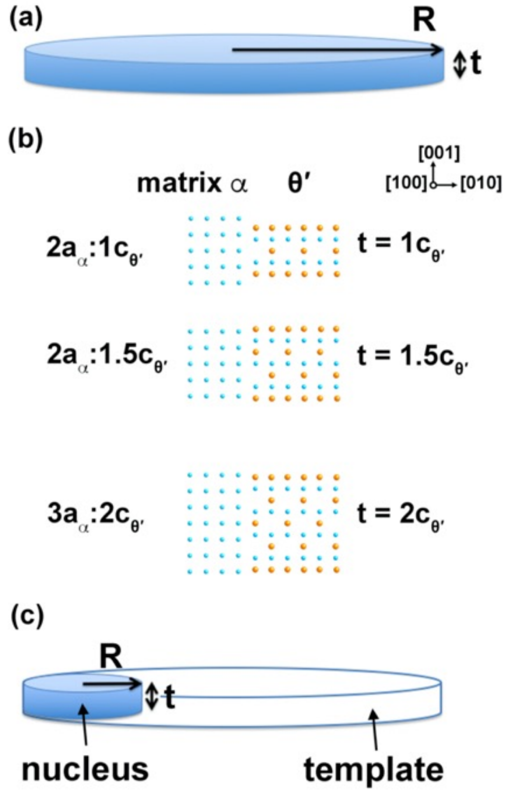

Supplementary Figure 16. Nucleus geometry and configurations. (a) The nucleus is described as a disk of radius R and thickness t. **(b)** The three smallest thicknesses t of θ′ phase are considered: $t = 1c_{\theta'}$, $t = 1.5c_{\theta'}$ and $t = 2c_{\theta'}$. **(c)** θ′ nucleus forming on a pre-existing template precipitate of similar thickness.

The chemical free energy ΔG_{chem} was estimated in three different ways.

(1) Under the assumption of a dilute and ideal solid solution, the chemical free energy ΔG_{chem} associated with nucleation of a phase β at temperature T can be approximated as

$$\Delta G_{chem} = -\frac{k_B T}{a_0} \ln \frac{x^{\alpha}_{Cu}}{x^{\beta}_{Cu}}, \qquad (3)$$

where k_B is the Boltzmann constant, a_0 is the volume per atom (assumed to be the same for matrix and precipitate, and equal to the atomic volume in aluminium, or a_0 = 16.6 Å^3), x^{α}_{Cu} the fraction of solute Cu atoms in the matrix before precipitation and x^{β}_{Cu} the fraction of solute Cu atoms at the solvus equilibrium at temperature T [5].

Using this method with the accepted metastable phase diagram for Al-Cu [6] we obtain $a_0 \Delta G^{\theta'}_{chem}$ = -61 meV per atom for θ′ nucleating at 160°C from a supersaturated solid solution of 1.7at.%Cu (*i.e.* x^{α}_{Cu} = 0.017) and $a_0 \Delta G^{\theta''}_{chem}$ = -36 meV per atom for θ″ nucleating in the same conditions.

Following nucleation of the θ″ phase at 160°C the supersaturation of the solid solution will be considerably reduced, to 0.65 at.%Cu according to the metastable solvus curves of the Al-Cu phase diagram [6]. This will result in a reduced chemical free energy of $a_0 \Delta G^{\theta'}_{chem}$ = -23 meV per atom.

(2) A more sophisticated form of ΔG_{chem} was proposed for the θ′ phase nucleating from the matrix based on a thermodynamic assessment [4] of the Al-Cu phase diagram [7]:

$$\Delta G_{chem} = G^{\beta}_{chem} - G^{\alpha}_{chem}, \qquad (4)$$

where G^{β}_{chem} and G^{α}_{chem} are the bulk free energies for the θ′ phase and the matrix, respectively, with

$$G^{\beta}_{chem} = \frac{-9245.8 + 0.579T}{96485 a_0} \qquad (5)$$

and

$$G^{\alpha}_{chem} = \frac{k_B T}{a_0}\left[x^{\alpha}_{Cu} \ln(x^{\alpha}_{Cu}) + (1 - x^{\alpha}_{Cu}) \ln(1 - x^{\alpha}_{Cu})\right] + \frac{x^{\alpha}_{Cu}(1 - x^{\alpha}_{Cu})}{96485 a_0}[(-24085 - 18.18944T) + (40399.8 - 3.91235T)(1 - 2x^{\alpha}_{Cu}) + (-19683 + 16.06993T)(-1 + 3(1 - 2x^{\alpha}_{Cu})^2)/2] \qquad (6)$$

in units of eV.m^{-3}.

Using this method we obtain $a_0 \Delta G_{chem}$ = -93 meV per atom for θ′ nucleating at 160°C from a supersaturated solid solution of 1.7at.%Cu.

No such equation has yet been proposed for the bulk free energy of the θ″ phase.

(3) The chemical free energy can also be estimated at 0K using our DFT calculations of the formation energy relative to the Cu defect energy (see right column of Supplementary Table 2); this, of course, does not include any entropic contribution (vibrational or configurational) and is therefore limited to the enthalpy term of ΔG_{chem}. Vaithyanathan *et al.* [8] calculated the chemical free energy using a combination of DFT, Monte Carlo and mixed-space cluster expansion simulations. The bulk free energy at 0K for the θ′ phase was calculated to be -196 meV per atom [8], which is

slightly less than our value of -180 meV per atom (see Supplementary Table 2). Vaithyanathan *et al.* [8] also found a significant vibrational entropy term, of ~27 meV at 160°C, but no significant configurational entropy contribution. Based on this work [8], $a_0 \Delta G_{chem}^{\theta'}$ = -155 meV per atom for θ′ nucleating at 160°C from a supersaturated solid solution of 1.7at.%Cu. This is relatively close to our 0K estimate of -140 meV per atom relative to the Cu defect energy, but is significantly larger than estimates obtained from the Al-Cu diagram (-61 meV and -93 meV, as shown in (1) and (2) above).

To estimate the elastic energy ΔG_{el} we use Christian's approximation for a coherent inclusion of oblate spheroidal shape [9], based on Eshelby's solution for an inclusion coherent with the matrix:

$$\Delta G_{el} = \frac{\mu}{(1-\upsilon)} \frac{\pi}{4} \varepsilon^2 \frac{t}{2R}, \qquad (7)$$

where R is the semi-major radius of the precipitate, t the semi-minor radius such that $t \ll R$, μ is the shear modulus (assumed to be the same for matrix and precipitate), ν Poisson's ratio and ε the tensile strain normal to the habit plane. We ignored the strain along the coherent direction (~0.0025) as it was found to have little effect on the calculations. The tensile/compressive strain ε was calculated using

$$\varepsilon = 2\frac{nc_{\theta'} - ma_{\alpha}}{nc_{\theta'} + ma_{\alpha}}, \qquad (8)$$

where n and m are the number of θ′ phase unit cells and corresponding number of matrix unit cells for a given precipitate thickness (see Supplementary Figure 16). This yields the following tensile/compressive strain values for the three thicknesses considered (Supplementary Figure 16): ε = -0.33 (for $t = 1c_{\theta'}$), ε = +0.07 (for $t = 1.5c_{\theta'}$) and ε = -0.05 (for $t = 2c_{\theta'}$).

The Poisson's ratio for aluminium of ν = 0.345 was used. For the shear modulus, μ, we chose values in the range of 28-90 GPa, which corresponds to that of pure aluminium (28 GPa) and the **θ′** phase according to DFT calculations at 0K (90 GPa) [10].

For the interfacial energies between the θ′ phase and the matrix we investigated the following range of values, based on the results from Refs. [10-14]: γ_c = 180-220 mJ.m^{-2} and γ_{sc} = 320-600 mJ.m^{-2}. The larger values for γ_{sc} correspond to the simple semi-coherent interface, whereas the lower value (320 mJ.m^{-2}) was obtained from a recent calculation [13] for the complex semi-coherent interface [11]. Regarding the coherent interface, experimental observations clearly reveal the presence of interstitial Cu atoms (see Fig. 2), in accordance with our earlier work [14]. Our calculations [13] yielded an interfacial energy of γ_c = 180 mJ.m^{-2} for the experimentally observed interface (*i.e.* that exhibiting interstitial Cu atoms) compared with 190 mJ.m^{-2} for the interface without interstitial atoms. Kim *et al.* [12] calculated a value of γ_c = 200 mJ.m^{-2} for the coherent interface without interstitial segregation, and reported a higher calculated interfacial energy for the experimentally observed interface, without providing values for this interfacial configuration.

Supplementary Note 3.1.1 Nucleation of θ′ phase directly from 1.7at.%Cu solid solution

Figure S17 shows the total energy change ΔG of a θ′ nucleus of radius R and thickness $t = 1c_{\theta'}$, $t = 1.5c_{\theta'}$ or $t = 2c_{\theta'}$ computed according to Supplementary Equation 2, for three different estimates of the chemical free energy $a_0\Delta G_{chem}$ for nucleation from a 1.7 at.%Cu solid solution (see Supplementary Note 3.1). In these plots the maximum of each curve corresponds to the energy barrier to nucleation, ΔG^*, and the critical radius, R^* (see Fig. 4(c)). In all cases, $t = 2c_{\theta'}$-thick nuclei are the preferred configuration. However it is clear that the different values of the chemical free energy obtained from different methods are associated with vastly different results for ΔG^* and R^*: according to our calculations, a $2c_{\theta'}$-thick nucleus could have a critical radius of 0.5 to 4 nm, depending on the chemical free energy used!

We also investigated the effect of different estimates for the shear modulus (Supplementary Figure 18), the coherent interfacial energy (Supplementary Figure 19) and the semi-coherent interfacial energy (Supplementary Figure 20). In these three cases, the semi-coherent interfacial energy is, not surprisingly, the most sensitive parameter on critical nucleus size and nucleation barrier. However the calculated changes in critical nucleus size and nucleation barrier for extreme estimates of the semi-coherent interfacial energy (320 and 600 mJ.m^{-2}), namely 1.5-2.5 nm and 2-6.10^{-18} J for a $2c_{\theta'}$-thick nucleus, are far less than the changes associated with the poorly known chemical free energy.

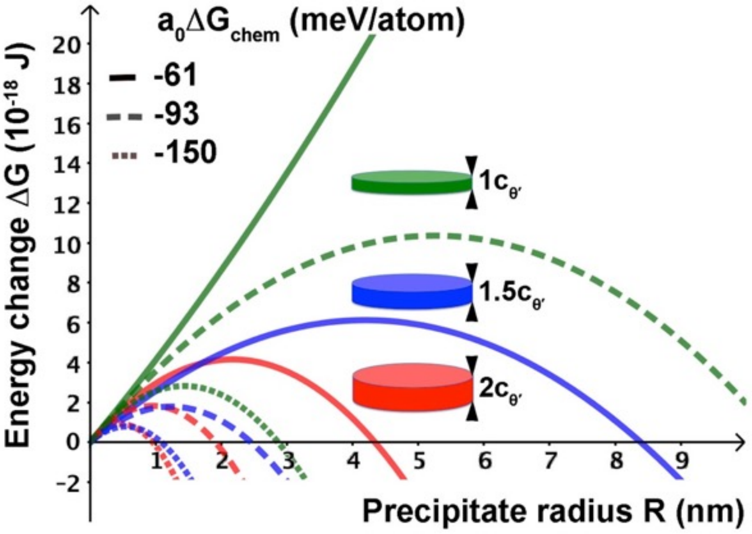

Supplementary Figure 17. Energy change of a θ′ nucleus of radius R and three different thicknesses ($t = 1c_{\theta'}$ – green curves –, $t = 1.5c_{\theta'}$ – blue curves –, and $t = 2c_{\theta'}$ – red curves), for three different estimates of the chemical free energy $a_0\Delta G_{chem}$. The chemical free energy has a large effect on the critical nucleus for nucleation and energy barrier. We used $\mu = 30$ GPa, $\gamma_c = 200$ mJ.m^{-2} and $\gamma_{sc} = 500$ mJ.m^{-2}.

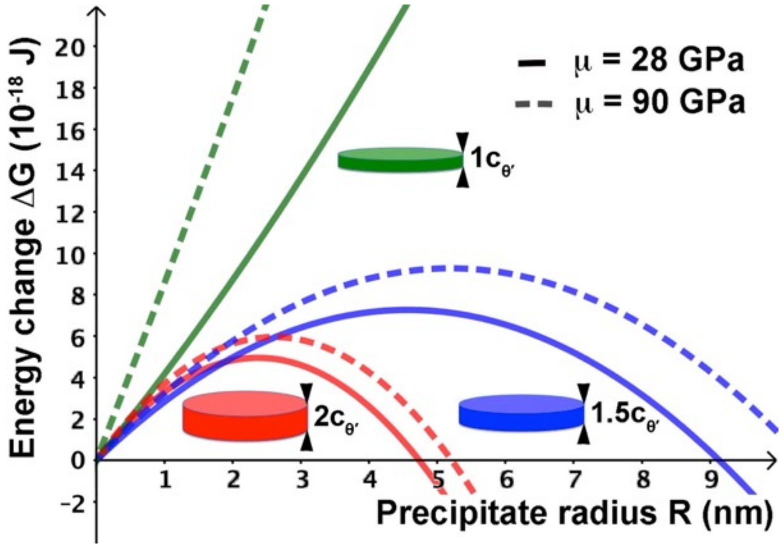

Supplementary Figure 18. Energy change of a θ′ nucleus of radius *R* and three different thicknesses (t = 1$c_{\theta'}$ – green curves –, t = 1.5$c_{\theta'}$ – blue curves –, and t = 2$c_{\theta'}$ – red curves), for two extreme values of the shear modulus, μ = 28 GPa and 90 GPa. These different values of the shear modulus do not markedly change the critical radius or the nucleation barrier. The values of γ_c = 200 mJ.m^{-2} , γ_{sc} = 500 mJ.m^{-2} and $a_0\Delta G_{chem}$= -61 meV per atom were used.

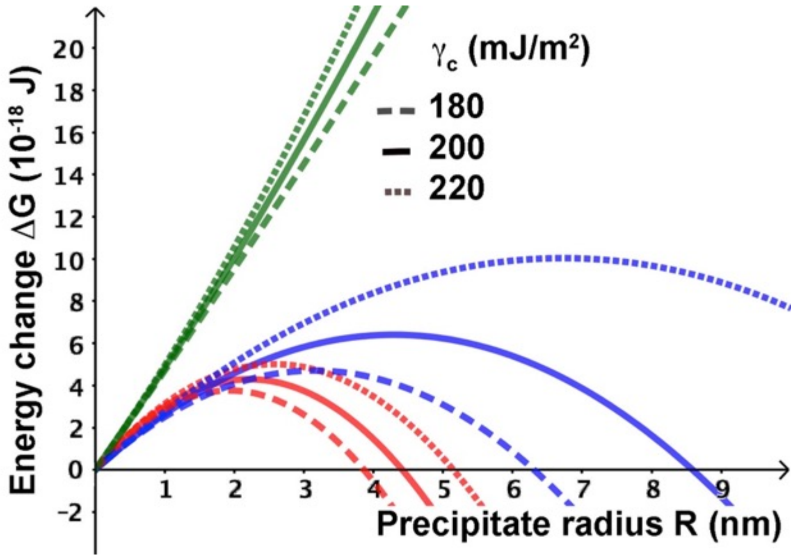

Supplementary Figure 19. Energy change of a θ′ nucleus of radius *R* and three different thicknesses (t = 1$c_{\theta'}$ – green curves –, t = 1.5$c_{\theta'}$ – blue curves –, and t = 2$c_{\theta'}$ – red curves), for three typical calculated values of the coherent interfacial energy, γ_c = 180 mJ.m^{-2}, γ_c = 200 mJ.m^{-2}, and γ_c = 220 mJ.m^{-2}. These different values of the coherent interfacial energy do not markedly change the critical radius or the nucleation barrier. The values of γ_{sc} = 500 mJ.m^{-2}, μ = 40 GPa and $a_0\Delta G_{chem}$= -61 meV per atom were used.

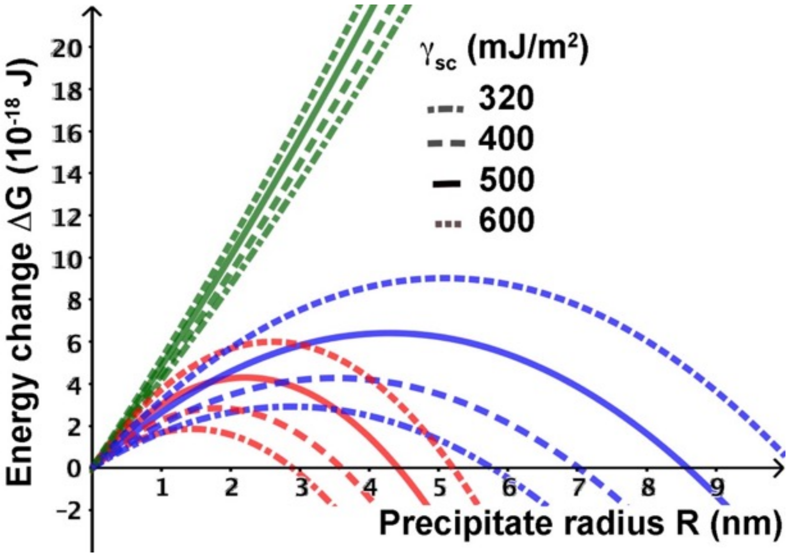

Supplementary Figure 20. Energy change of a θ′ nucleus of radius *R* and three different thicknesses (*t* = 1$c_{\theta'}$ – green curves –, *t* = 1.5$c_{\theta'}$ – blue curves –, and *t* = 2$c_{\theta'}$ – red curves), for four values of the semi-coherent interfacial energy, γ_{sc} = 320 mJ.m^{-2}, γ_{sc} = 400 mJ.m^{-2}, γ_{sc} = 500 mJ.m^{-2}, and γ_{sc} = 600 mJ.m^{-2}. These different values of the semi-coherent interfacial energy resulted in significantly different values of the critical radius and nucleation barrier. The values of γ_c = 200 mJ.m^{-2}, μ = 40 GPa and $a_0\Delta G_{chem}$= -61 meV per atom were used.

Based on the above results, chemical free energy is the most sensitive and least accurately known parameter in the CNT calculations. Supplementary Equation 3 yielded values of the critical radius and thickness that were closest to the experimental values. In addition, Supplementary Equation 3 provides a way to estimate the chemical free energy for the θ″ phase. We will therefore use Supplementary Equation 3 for all subsequent calculations, as well as the following values: μ = 60 GPa, γ_c = 200 mJ.m^{-2} and γ_{sc} = 500 mJ.m^{-2}. Our choice for γ_c is the average value of previous studies [8-12]. Regarding γ_{sc}, we selected the average value for the simple semi-coherent interfacial structure. The main reason for this is that TDN of θ′ does not appear to involve the previously observed complex interfacial structure [11]. In addition, the structure and energetics of this complex interface have not been fully clarified [11,13].

Supplementary Note 3.1.2 Nucleation of θ′ phase directly from 0.65at.%Cu solid solution

This condition corresponds to the remaining supersaturation of the solid solution following nucleation of the θ″ phase. In this situation the chemical free energy is reduced to $a_0\Delta G_{chem}^{\theta'} =$ -23 meV per atom (see Supplementary Note 3.1). Using the same interfacial energies and elastic constants as in Supplementary Figure 17, we obtain the plots shown in Supplementary Figure 21. According to our simple CNT model, homogeneous nucleation of the θ′ phase cannot take place in this reduced supersaturation of the solid solution, as the total energy change for nucleation never becomes negative. Therefore at a temperature below the θ″ solvus, such as the 160°C considered here, only heterogeneous nucleation of the θ′ phase appears possible.

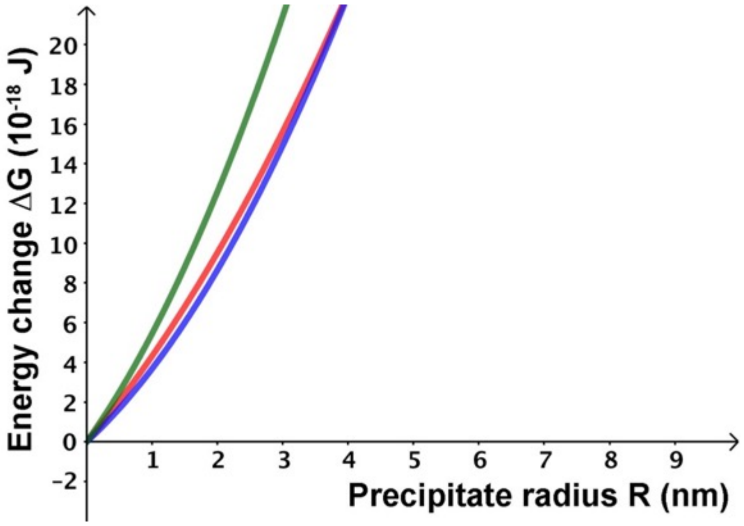

Supplementary Figure 21. Energy change of a θ′ nucleus of radius R and three different thicknesses ($t = 1c_{\theta'}$ – green curves –, $t = 1.5c_{\theta'}$ – blue curves –, and $t = 2c_{\theta'}$ – red curves), for the case of reduced supersaturation following nucleation of the θ″ phase. None of the energy curves become negative, implying that homogeneous nucleation of the θ′ phase is not possible. The values of μ = 40 GPa, γ_c = 200 mJ.m^{-2}, γ_{sc} = 500 mJ.m^{-2} and $a_0\Delta G_{chem}$= -23 meV per atom were used.

Supplementary Note 3.2. Template directed nucleation in pure Al-Cu

The geometry of a θ′ nucleus forming on a template θ″ precipitate is shown Supplementary Figure 16(c). To model template-directed nucleation (TDN), we simply modify Supplementary Equation 2 so as to estimate the difference in energy, ΔG_{TDN}, between a section of θ″ phase replaced by a θ′ nucleus of radius R:

$$\Delta G_{TDN} = V(\Delta G_{chem} + \Delta G_{el}) + 2\pi R t \gamma_{sc} , \qquad (9)$$

where ΔG_{chem} is now the difference in chemical energy between θ″ and θ′, and ΔG_{el} the difference in elastic energy between the two phases, namely $\Delta G_{el} = \Delta G_{el}^{\theta'} - \Delta G_{el}^{\theta''}$. The elastic energies are calculated using Supplementary Equation 7, with ε = -0.04 for the θ″ phase and the relevant values for each corresponding θ′ precipitate thickness (see Supplementary Note 3.1). Here we assume that there is no change in coherent interfacial energy due to templating, even for $2c_{\theta'}$-thick precipitates. In other words, the total contribution of the coherent interfacial energy is 0. Also assumed is that the semi-coherent interfacial energy of the nucleus is constant around its perimeter, even though this interface will be shared with the matrix or θ″ (see Supplementary Figure 16(c)).

Supplementary Figure 22 shows a comparison between the total energy change associated with TDN and SSSN (homogeneous nucleation from a 1.7at.% supersaturated solid solution, as in Supplementary Figure 17 for $a_0\Delta G_{chem}$=-61 meV per atom). We can see that TDN is as favourable as SSSN. More importantly, even in reduced solute supersaturation, TDN is possible, in contrast to homogeneous nucleation (Supplementary Figure 21). Furthermore the $t = 1.5c_{\theta'}$ configuration becomes very slightly favoured over $t = 2c_{\theta'}$ both in terms of the barrier to nucleation and the critical number of atoms N^*, since $N^* = 2\pi R^{*2} t$, with $R^*(t = 1.5c_{\theta'})$ = 2.5 nm and $R^*(t = 2c_{\theta'})$ = 2.2 nm and hence $N^*(t = 1.5c_{\theta'})/N^*(t = 2c_{\theta'})$ ~0.95.

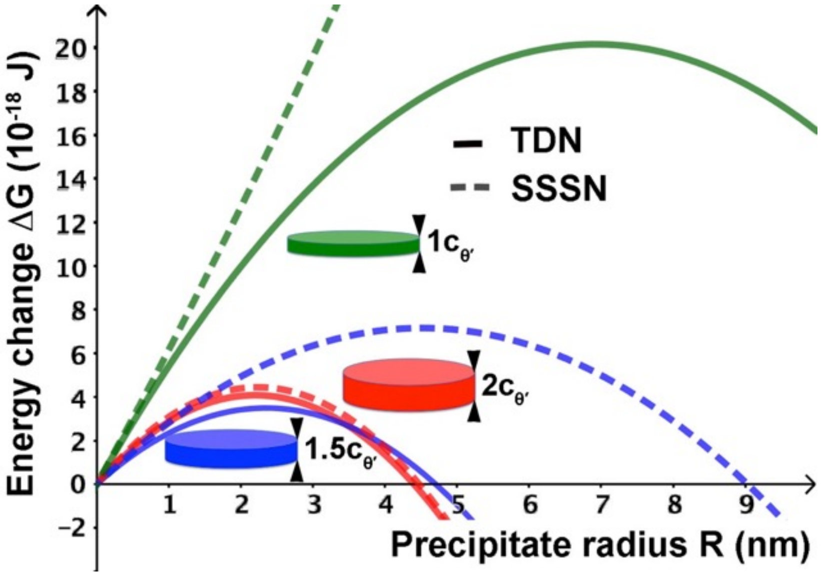

Supplementary Figure 22. Energy change of a θ′ nucleus of radius *R* and three different thicknesses (t = 1$c_{\theta'}$ – green curves –, t = 1.5$c_{\theta'}$ – blue curves –, and t = 2$c_{\theta'}$ – red curves), for template-directed nucleation of θ′ on the θ″ phase (TDN, solid curves) and homogeneous nucleation of θ′ from a 1.7at.% supersaturated solid solution (SSSN, dashed curves). Despite the reduced supersaturation, TDN is possible, with energy barriers and critical nuclei of a similar magnitude to SSSN. In contrast to SSSN (Supplementary Figure 12), TDN slightly favours 1.5$c_{\theta'}$ -thick nuclei. The values of μ = 60 GPa, γ_c = 200 mJ.m^{-2} , γ_{sc} = 500 mJ.m^{-2} and $a_0\Delta G_{chem}$= -24 meV per atom were used.

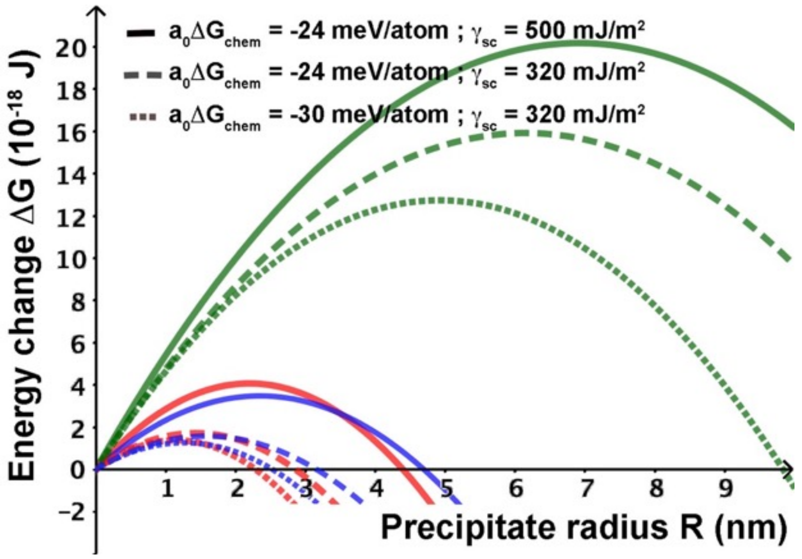

Supplementary Figure 23. Energy change of a θ′ nucleus of radius *R* and three different thicknesses (t = 1$c_{\theta'}$ – green curves –, t = 1.5$c_{\theta'}$ – blue curves –, and t = 2$c_{\theta'}$ – red curves), for TDN of θ′ on the θ″ phase in pure Al-Cu for three different combinations of the two most sensitive parameters, the volume free energy and semi-coherent interfacial energy. The t = 1.5$c_{\theta'}$ and t = 2$c_{\theta'}$ thicknesses remain equally favourable for the three combinations of parameters, with nearly unchanged barriers to nucleation. However the critical radius for nucleation is quite sensitive to the set of parameters used.

We examined how the nucleation barrier and critical radius for TDN of the θ′ phase vary as a function of the two most sensitive parameters, the volume free energy and semi-coherent interfacial energy. Supplementary Figure 23 shows plots of the total energy change associated with nucleation for three combinations of $a_0\Delta G_{chem}$ and γ_{sc}. It can be seen that the t = 1.5$c_{\theta'}$ and t = 2$c_{\theta'}$ thicknesses remain equally favourable for the three combinations of parameters, with nearly unchanged barriers to nucleation. In contrast, the critical radius for these two thicknesses changes significantly, from ~2.5 nm to ~1.3 nm for both lower volume free energy and lower interfacial energy. These changes are not surprising in the light of our above calculations for nucleation from the supersaturated solid solution (see Supplementary Figure 17 and Supplementary Figure 21). However they still predict a critical radius for nucleation greater than the observed value (~1.3 nm versus ~ 1 nm), even with the most beneficial values of the volume free energy and semi-coherent interfacial energy. In an attempt to resolve this discrepancy between our experiments and CNT calculations, an additional contribution to the total free energy will be presented in Supplementary Note 3.4.

Supplementary Note 3.3. Template-directed nucleation in Al-Cu microalloyed with Au

Chen *et al.*'s recent work [15] reported that Au atoms substitute for Cu atoms in the θ′ phase and enhance its precipitation rate, including that of single unit cell thick precipitates. DFT calculations [15] found that Au substitution of Cu in θ′ will lower the chemical free energy. Based on this work [15], a value of $a_0\Delta G_{chem}$= -40 meV per atom (instead of $a_0\Delta G_{chem}$= -24 meV per atom for the pure Al-Cu case) seems reasonable. Also including a reduction in tensile strain for t = 1$c_{\theta'}$ and t = 2$c_{\theta'}$ thicknesses of ε = -0.17 and ε = 0, respectively (see Supplementary Figure 24), results in nucleation barriers and critical radii that are consistent with observations. In particular, the barrier for nucleation is roughly the same for all three thicknesses. Although the critical radius is ~ 2 nm for t = 1$c_{\theta'}$ compared with ~1.3 nm for t = 2$c_{\theta'}$, the thinner configuration will be associated with a lower critical number of atoms. Note that the value chosen for the tensile strain does not affect the t = 2$c_{\theta'}$ curve much, in contrast to the t = 1$c_{\theta'}$ case.

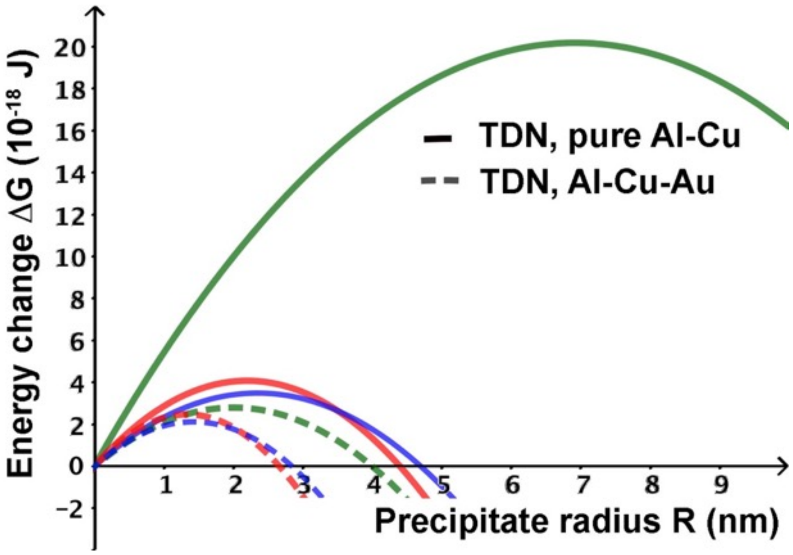

Supplementary Figure 24. Energy change of a θ′ nucleus of radius *R* and three different thicknesses (t = 1$c_{\theta'}$ – green curves –, t = 1.5$c_{\theta'}$ – blue curves –, and t = 2$c_{\theta'}$ – red curves), for TDN of θ′ on the θ″ phase in pure Al-Cu (solid curves) and TDN of θ′ on the θ″ phase in Al-Cu-Au (dashed curves). The replacement of 50% of Cu atoms within θ′ by Au atoms is modelled by a decrease in the volume free energy ($a_0 \Delta G_{chem}$= -40 meV per atom) and a decrease in the positive tensile strain for t = 1$c_{\theta'}$ and t = 2$c_{\theta'}$ configurations (ε = -0.17 and ε = 0, respectively). Single unit cell-thick precipitates are now as likely to nucleate as thicker configurations.

Supplementary Note 3.4. Template directed nucleation with thermodynamic assistance from vacancies

We noted above that TDN near the surface of a thin Al-Cu sample exhibits a critical radius of nucleation significantly smaller (1) than observed for nucleation in the bulk (see Supplementary Figure 9) and (2) than predicted by CNT. These two observations strongly suggest that TDN in Al-Cu is enabled by a factor that is specific to proximity to the surface. Since the semi-coherent interfacial energy for a precipitate in contact with the aluminium oxide layer at the sample surface is unlikely to be much lower than that in bulk aluminium, the only other possible factors are vacancies. Vacancies no doubt play a major role in enhancing the kinetics of nucleation. The reduced critical radius further shows that vacancies must also exert a thermodynamic effect on TDN.

Vacancies can lower the critical radius and energy barrier by accommodating the transformation strain of nucleation. This transformation strain comprises volumetric strain resulting from nucleation of a precipitate occupying a volume greater than that of the matrix, as well as shear strain, resulting from the shape change associated with nucleation. Whereas volumetric strain will always remain after nucleation (this is residual misfit strain), shape strain can in part be absorbed by the semi-coherent interfacial energy, through a/2<100> misfit dislocations [11]. Of the three lowest θ′ precipitate thicknesses only 1.5$c_{\theta'}$ can have their volumetric strain accommodated by vacancies since the misfit strain values are ε = +0.07 for t = 1.5$c_{\theta'}$ but ε = -0.33 for t = 1$c_{\theta'}$, and ε = -0.05 for t = 2$c_{\theta'}$. The shape change corresponds to the (001) plane stacking change, from ABA in the matrix, to AAA in θ′: vacancies can accommodate the compression caused by this stacking change (see Fig. 4(b) and Supplementary Figure 13).

To quantify the energy gained from vacancies accommodating the transformation strain of nucleation, we adapt the treatment proposed by Russell [14] and Marth *et al.* [17]. The total energy change of the system can be written as:

$$\Delta G_{TDN}^{V} = V(\Delta G_{chem} + \Delta G_{chem}^{V} + \Delta G_{el}^{V}) + 2\pi R t \gamma_{sc}^{V}. \qquad (10)$$

Here ΔG_{el}^{V} is the modified volumetric strain energy of the nucleus resulting from vacancy strain accommodation, ΔG_{chem}^{V} is the chemical free energy resulting from the net change in vacancy numbers in the matrix due to strain accommodation, and γ_{sc}^{V} is the modified semi-coherent interfacial energy arising from vacancy accommodation.

For the sake of simplicity, we assume that ΔG_{el}^{V} is unchanged for t = $1c_{\theta'}$ and t = $2c_{\theta'}$, and 0 for t = $1.5c_{\theta'}$. In other words, vacancies are assumed to be in sufficient numbers to fully accommodate the volumetric strain of a $1.5c_{\theta'}$-thick precipitate. For a misfit strain value of ε = +0.07 (*i.e.* for t = $1.5c_{\theta'}$), this corresponds to a volume of approximately one vacancy for every 7 surface atoms. Despite the crudeness of this model, this value is very close to the maximum number of vacancies that will be favoured to segregate at the coherent interfaces of a θ′ precipitate, namely 3 vacancies per 20 surface atoms (see Supplementary Table 3).

Removing vacancies from an equilibrium vacancy population in the matrix in order to accommodate strain around the nucleus will result in a chemical energy cost partly offsetting the gain in strain energy. This situation will lead to little change to the nucleation barrier. However, the presence of excess vacancies will ensure significant gains in chemical free energy ΔG_{chem}^{V}. The number of excess vacancies can be described by the vacancy supersaturation

$$Vss = C_v/C_{eq}, \qquad (11)$$

where C_{eq} is the vacancy equilibrium concentration at temperature T and C_v is the actual vacancy concentration. In the bulk of the sample, *Vss* will be ~1 given that most quenched-in vacancies will have been lost to sinks following ageing for 24 h at 160°C. However very close to the surface vacancy concentrations as high as 10 orders of magnitude greater than C_{eq} may be possible [18]. Such enormous supersaturations can be obtained by assuming that most surface-generated thermal vacancies diffuse into the first 1 nm below the surface. The equilibrium vacancy concentrations can be calculated via the well-known Arrhenius equation

$$C = e^{-\frac{E_f}{k_B T}}, \qquad (12)$$

where E_f is the vacancy formation energy. In bulk aluminium E_f = 0.65 eV, but at the surface the vacancy formation energy can be as low as E_f^S= 0.15 eV [19]. An upper bound for the natural logarithm of the vacancy supersaturation just below the sample surface can be therefore estimated as

$$ln(V_{SS}) = \frac{E_f - E_f^S}{k_B T}. \qquad (13)$$

For E_f^S = 0.25 eV and 0.45 eV, $ln(V_{SS})$ =10.7 and 5.4, respectively.

The energy gain associated with vacancies accommodating misfit strain can be written as an additional term to the volume free energy:

$$\Delta G_{chem}^{V} = - k_B T . ln(V_{SS}) \left[\frac{V\varepsilon}{a_0} + \frac{\pi R}{2a_\alpha}\right] \qquad (14)$$

Here the first term in the square bracket corresponds to the volume that needs to be accommodated (remember that V is the volume of the nucleus and ε its misfit strain); the second term is the linear area subjected to compression around the nucleus' perimeter as a result of the ABA $\rightarrow$ AAA stacking change. a_α is the lattice parameter of aluminium. Our DNNP simulations (Supplementary Figure 14 and Supplementary Table 3) suggest segregation of one vacancy per $2a_\alpha$ distance along half the semi-coherent interface. Clearly this is an approximation and much more extensive and accurate calculations are required to determine the optimum number and configuration of vacancies at the semi-coherent interface. Similarly, it is difficult to estimate the effect of vacancy segregation on the semi-coherent interfacial energy γ_{sc}^{V}. Previous authors have suggested that the structural component of interfacial energy is the dominant component [20]. Therefore in the following we will investigate the effect of a significant reduction in γ_{sc}^{V} on the nucleation barrier.

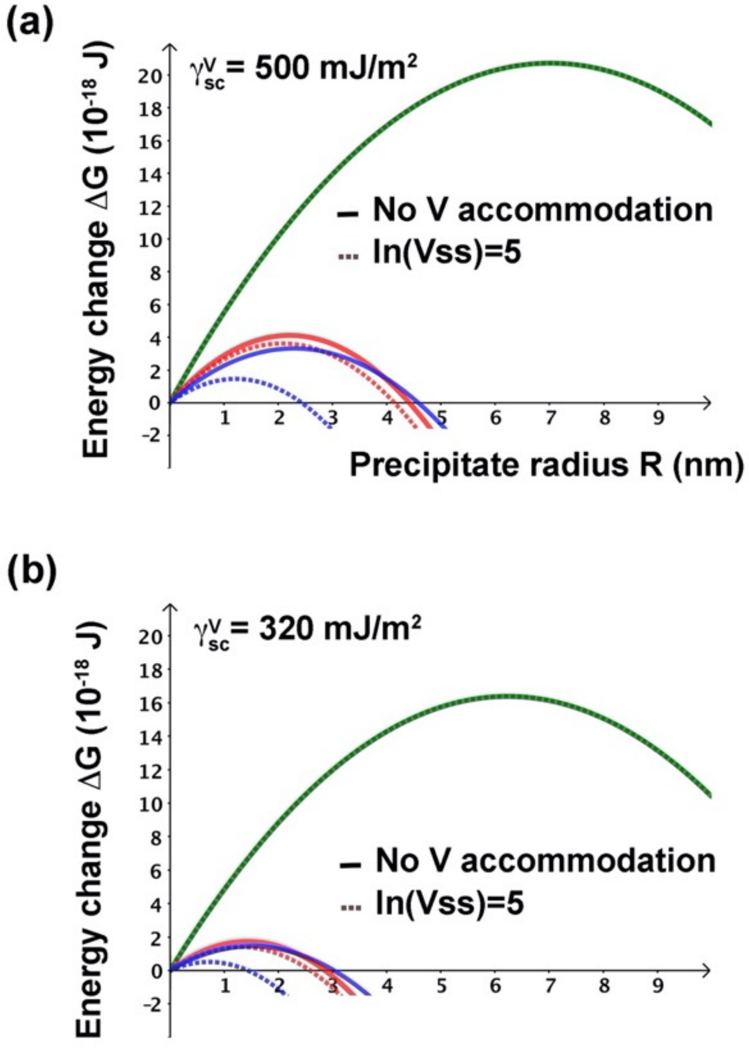

Supplementary Figure 25. Energy change of a θ′ nucleus of radius *R* for two different vacancy supersaturations *Vss*, shown as ln(Vss), and two values of the semi-coherent interfacial energy for TDN of θ′ on the θ″ phase in pure Al-Cu. Vacancies are modelled to accommodate both the residual misfit strain and the shape misfit. **(a)** γ_{sc} = 500 mJ.m^{-2} and **(b)** 320 mJ.m^{-2}. A value of $a_0 \Delta G_{chem}$= -24 meV per atom was used. The energy barrier and critical radius of nucleation can be substantially reduced through the accommodation of volumetric misfit strain and shape strain by vacancies.

Plots of the total change in energy associated with TDN, including the two vacancy contributions just described, are displayed in Supplementary Figure 25, for two values of the semi-coherent interfacial energy γ_{sc}^{V} and for a reasonably small value of the vacancy supersaturation V_{ss} (lnV_s =5). It can be seen that strain accommodation by vacancies results in a substantial lowering of both the energy barrier and the critical radius. The latter, in particular, now reaches values closer to the experimentally measured 1 nm.

Both the residual misfit volumetric strain and shape strain (*i.e.* change in {002} stacking) contributions of vacancy accommodation result in a similar reduction in the energy barrier and critical radius, as shown in Supplementary Figure 26. However different precipitate thicknesses will be affected differently: volumetric strain accommodation will only assist t = 1.5$c_{\theta'}$ nuclei (Supplementary Figure 26(a)), whereas shape strain accommodation will assist both t = 1.5$c_{\theta'}$ and t = 2$c_{\theta'}$ nuclei (Supplementary Figure 26(b)).

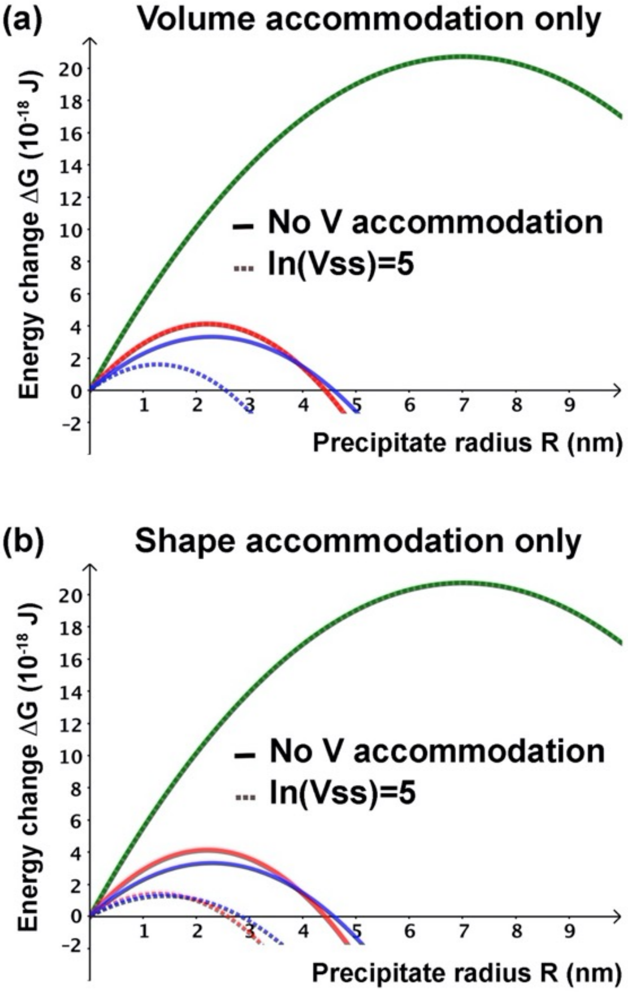

Supplementary Figure 26. Energy change of a θ′ nucleus of radius *R* for two different vacancy supersaturations Vss, shown as ln(Vss), and for the two possible mechanisms of vacancy accommodation: (a) volume strain accommodation and (b) shape strain accommodation, for TDN of θ′ on the θ″ phase in pure Al-Cu. Values of $a_0 \Delta G_{chem}$= -24 meV per atom and γ_{sc} = 500 mJ.m^{-2} were used. The energy barrier and critical radius of nucleation can be substantially reduced, by similar amounts, through the accommodation of volumetric misfit strain or shape strain by vacancies. Not surprisingly, volume accommodation favours 1.5$c_{\theta'}$-thick nuclei.

Supplementary Note 3.4. Nucleation directly from SSS, with thermodynamic assistance from vacancies

Vacancies can also assist nucleation of the θ′ phase directly from the supersaturated solid solution. Calculation of the thermodynamic barrier to nucleation for this case involves adding Supplementary Equation 14 to Supplementary Equation 2 and assuming full accommodation of the elastic strain energy, i.e. ΔG_{el} =0, as in Supplementary Note 3.4. The vacancy supersaturation will be significantly larger, as expected from quenching the alloy from the solution treatment temperature (525°C). Without loss of vacancies during quenching and ageing at 160°C, the supersaturation is ln(Vss)=7.9. Assuming half the excess vacancies are lost (e.g. to grain boundaries), ln(Vss)=7, which is the value used for the plots shown in Supplementary Figure 27. Not surprisingly, vacancy accommodation significantly reduces the nucleation barrier and critical radius for 1.5$c_{\theta'}$-thick nuclei. However, these values remain higher than for TDN, as can be seen by comparing with Supplementary Figure 25(a). In addition, nucleation of the coherent phase θ″ should remain favoured over nucleation of θ′, as the former is controlled by diffusion of Cu solute rather than interfacial energy.

The fact that, once formed, θ″ does not appear to transform to θ′ via TDN in conventional heat treatments, may be attributed to an insufficient number of vacancies. This may be due to the slow kinetics of the formation of the θ″ phase, as evident from hardness curves in an early study [21]: the aggregation of solute Cu into θ″ precipitates may be too slow to retain the vacancies associated with diffusing solute and to enable the critical number of vacancies required for θ′ nucleation.

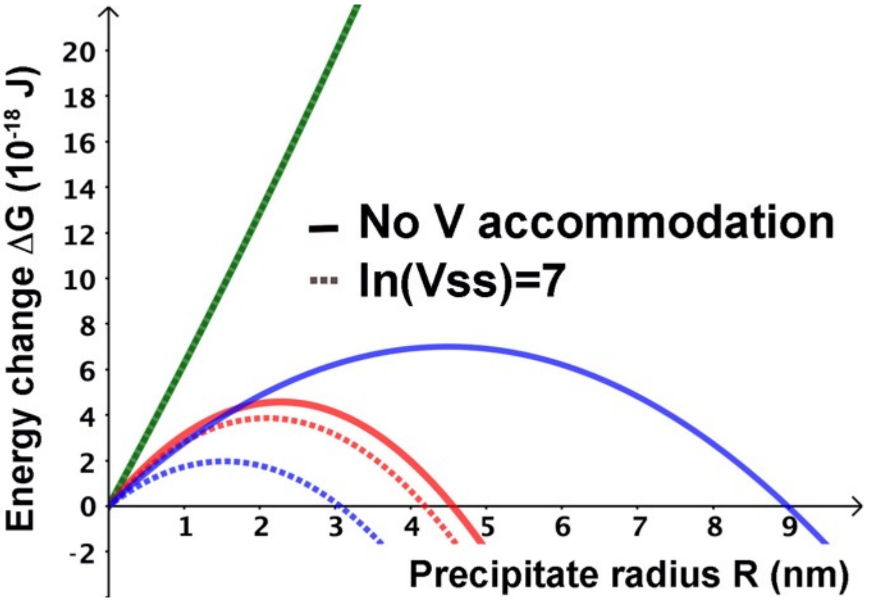

Supplementary Figure 27. Energy change of a θ′ nucleus of radius R for two different vacancy supersaturations Vss, shown as ln(Vss), for nucleation of θ′ directly from the supersaturated solid solution (SSS) in pure Al-Cu. Values of $a_0\Delta G_{chem}$= -61 meV per atom, γ_c = 200 mJ.m^{-2} , γ_{sc} = 500 mJ.m^{-2} and μ = 60 GPa were used. The critical radius of nucleation is reduced significantly for 1.5$c_{\theta'}$-thick nuclei, but remain higher than for TDN (compare with Supplementary Figure 25(a)).

Supplementary References

1. P.P. Ma, C.H. Liu, Z. Ma, L. Zhan, M. Huang, Formation of a new intermediate phase and its evolution toward θ′ during aging of pre-deformed Al-Cu alloys, J. Mater. Sci. Tech. 35, 885-890 (2019).
2. C. Liu, P. Ma, L. Zhan, M. Huang, Multiple precipitation reactions and formation of θ′-phase in a pre-deformed Al-Cu alloy, Mater. Sci. Eng. A 733, 28-38 (2018).
3. U. Dahmen, K.H. Westmacott, Ledge structure and the mechanism of θ′ precipitate growth in Al-Cu, Phys. Stat. Sol. A 80, 249-262 (1983) .
4. M.V. Petrik, Y.N. Gornostyrev, P.A. Korzhavyi, Scripta Mater. 165, 123-127 (2019).
5. D.A. Porter, K.E. Easterling, Phase Transformations in Metals and Alloys (CRC Press, 2nd Edition, 1992).
6. J.L. Murray, The aluminium-copper system, Int. Met. Rev. 30, 211-233 (1985).
7. S.Y. Hu, Phase-field models of microstructure evolution in a system with elastic inhomogeneity and defects, Ph.D. thesis, Pennsylvania State University, 2004.
8. V. Vaithyanathan, C. Wolverton, L.Q. Chen, Multiscale modeling of θ′ precipitation in Al-Cu binary alloys, Acta Mater. 52, 2973-2987 (2004).
9. J.W. Christian, Accommodation strains in martensite formation, and the use of a dilation parameter, Acta Metall. 6, 377-379 (1958).
10. S.Y. Hu, M.I. Baskes, M. Stan, L.Q. Chen, Atomistic calculations of interfacial energies, nucleus shape and size of θ′ precipitates in Al-Cu alloys, Acta Mater. 54, 4699-4707 (2006).
11. L. Bourgeois, N.V. Medhekar, A.E. Smith, M. Weyland, J.F. Nie, C. Dwyer, Efficient atomic-scale kinetics through a complex heterophase interface, Phys. Rev. Lett. 111, 046102 (2013).
12. K. Kim, A. Roy, M.P. Gururajan, C. Wolverton, P.W. Voorhees, First-principles/Phase-field modeling of θ′ precipitation in Al-Cu alloys, Acta Mater. 140, 344-354 (2017).
13. Y. Zhang, Precipitation pathways in Al-Cu-(In-Sb)/Ge alloys, Ph.D. thesis, Monash University, 2019.
14. L. Bourgeois, C. Dwyer, M. Weyland, J.F. Nie, B.C. Muddle, Structure and energetics of the coherent interface between the θ′ precipitate phase and aluminium in Al-Cu, Acta Mater. 59, 7043-7050 (2011).
15. Y. Chen, Z. Zhang, Z. Chen, A. Tsalanidis, M. Weyland, S. Findlay, L.J. Allen, J. Li, N.V. Medhekar, L. Bourgeois, The enhanced theta-prime (θ′) precipitation in an Al-Cu alloy with Au additions, Acta Mater. 125, 340-350 (2017).
16. K.C. Russell, The role of excess vacancies in precipitation, Scripta Metall. 3, 313-316 (1969).
17. P.E. Marth, H.I. Aaronson, G.W. Lorimer, T.L. Bartel, K.C. Russell, Application of heterogeneous nucleation theory to precipitate nucleation at GP zones, Met. Trans. A 7, 1519-1528 (1976).
18. Q.S. Mei, K. Lu, Melting of metals: role of concentration and migration of vacancies at surfaces, Phil. Mag. Lett. 88, 203-211 (2008).
19. S.S. Gupta, M.A. van Huis, M. Dijkstra, M.H.F. Sluiter, Depth dependence of vacancy formation energy at (100), (110), and (111) Al surfaces: a first-principles study, Phys. Rev. B 93, 085432 (2016).
20. H.I. Aaronson, J.B. Clark, C. Laird, Interfacial energy of dislocation and of coherent interphase boundaries, Met. Sci. J. 2, 155-158 (1968).
21. H.K. Hardy, The ageing characteristics of ternary aluminium-copper alloys with cadmium, indium, or tin, J. Inst. Metals 80, 483-492 (1951-52).
